# Supplementary material for: Physical activity and self-rated health during retirement transition: a multitrajectory analysis of concurrent changes among public sector employees
Source: BMJ Open. 2023 Sep 29;13(9):e073876. doi: 10.1136/bmjopen-2023-073876 (PMC10546116; doi:10.1136/bmjopen-2023-073876)

Supplement 1. Trajectories of physical activity and self-rated health. All the groups are presented within the same scale.

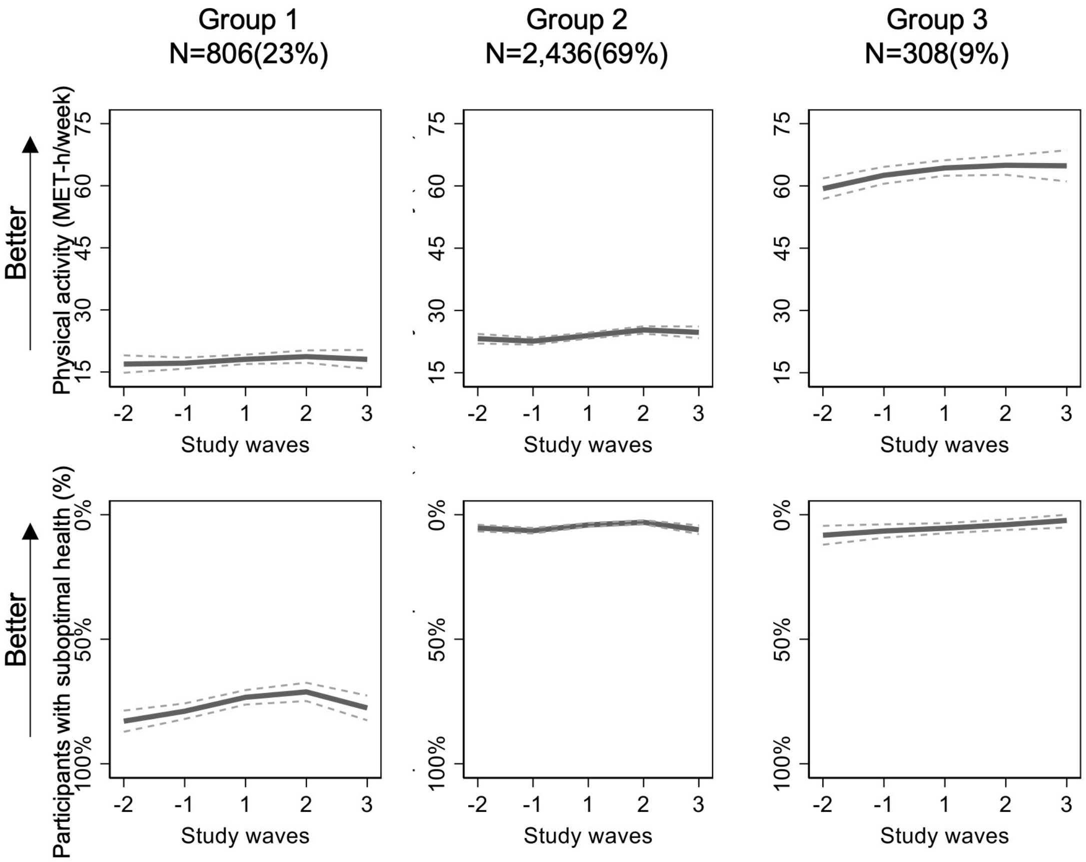

Supplement: Supplementary data [file bmjopen-2023-073876supp001.pdf]
