# Supplementary material for: Physical activity and self-rated health during retirement transition: a multitrajectory analysis of concurrent changes among public sector employees
Source: BMJ Open. 2023 Sep 29;13(9):e073876. doi: 10.1136/bmjopen-2023-073876 (PMC10546116; doi:10.1136/bmjopen-2023-073876)
Supplement: Supplementary data [file bmjopen-2023-073876supp002.pdf]

Supplement 2. The level of physical activity and physical functioning at different waves by trajectory groups

Study waves	Group #1:			Group #2:			Group #3:		
	Estimated mean	95% CI		Estimated mean	95% CI		Estimated mean	95% CI	
Physical activity, MET-h/week									
-2	16.9	14.8	19.0	23.2	22.1	24.4	59.4	56.9	61.8
-1	17.1	15.8	18.5	22.6	21.8	23.5	62.6	60.5	64.6
1	18.1	16.9	19.2	24.0	23.2	24.7	64.3	62.5	66.2
2	18.7	17.2	20.2	25.3	24.4	26.2	65.0	62.7	67.3
3	18.1	15.8	20.3	24.7	23.3	26.1	64.8	61.1	68.6
Percentage of participants with suboptimal self-rated health, %									
-2	83	79	87	5	4	7	8	4	12
-1	79	76	82	6	5	8	7	4	9
1	73	70	76	4	3	5	5	3	7
2	71	67	75	3	2	4	4	2	6
3	78	73	83	6	4	8	2	0	5
