# Supplementary material for: Physical activity and self-rated health during retirement transition: a multitrajectory analysis of concurrent changes among public sector employees
Source: BMJ Open. 2023 Sep 29;13(9):e073876. doi: 10.1136/bmjopen-2023-073876 (PMC10546116; doi:10.1136/bmjopen-2023-073876)
Supplement: Supplementary data [file bmjopen-2023-073876supp003.pdf]

Supplement 3.

Trajectories of physical activity and self-rated health, self-rated health treated as a continuous variable. The 95% CI:s are presented as dash lines in the figure.

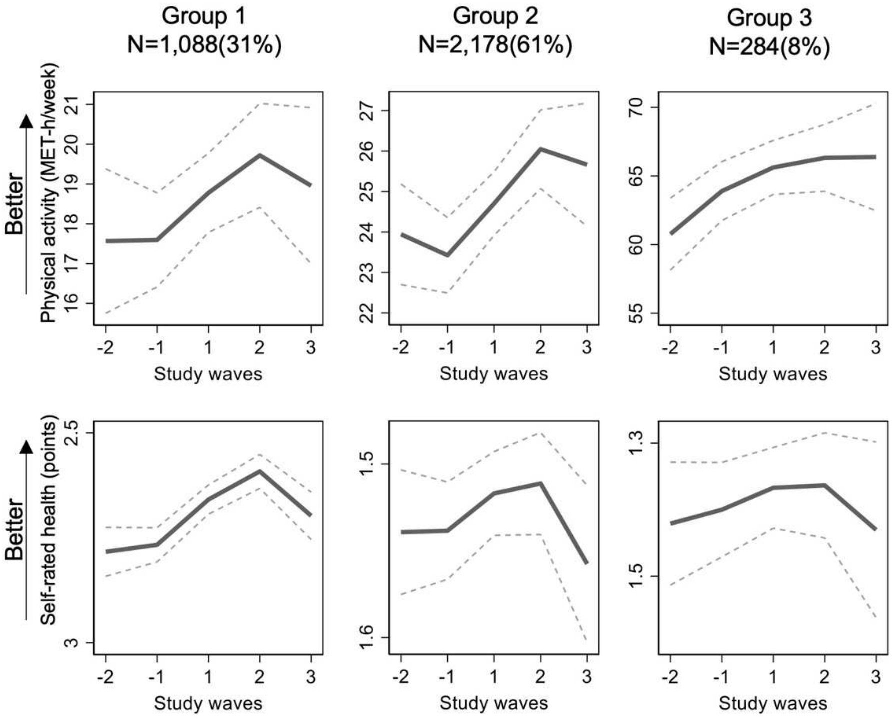

Goodness of fit of group-based trajectory analysis models, self-rated health treated as a continuous variable. The chosen model is shown in bold.

Number of clusters	Shape of trajectory	Smallest group		BIC[1]	AIC[2]	Smallest APP[3]
		N	%			
1	Cubic	3,550	100	75,810	76,769	1.0
2	Cubic	1,453	41	72,792	72,714	0.93
**3**	**Cubic**	**284**	**8**	**71,224**	**71,109**	**0.93**
4	Cubic	226	6	70,213	70,062	0.92

[1] BIC = Bayesian Information Criterion, [2] AIC = Akaike information criterion, [3] APP = Smallest average posterior probability
