# Supplementary material for: Physical activity and self-rated health during retirement transition: a multitrajectory analysis of concurrent changes among public sector employees
Source: BMJ Open. 2023 Sep 29;13(9):e073876. doi: 10.1136/bmjopen-2023-073876 (PMC10546116; doi:10.1136/bmjopen-2023-073876)
Supplement: Author's manuscript [file bmjopen-2023-073876.draft_revisions.pdf]

BMJ Open

BMJ Open is committed to open peer review. As part of this commitment we make the peer review history of every article we publish publicly available.

When an article is published we post the peer reviewers' comments and the authors' responses online. We also post the versions of the paper that were used during peer review. These are the versions that the peer review comments apply to.

The versions of the paper that follow are the versions that were submitted during the peer review process. They are not the versions of record or the final published versions. They should not be cited or distributed as the published version of this manuscript.

BMJ Open is an open access journal and the full, final, typeset and author-corrected version of record of the manuscript is available on our site with no access controls, subscription charges or pay-per-view fees (http://bmjopen.bmj.com).

If you have any questions on BMJ Open's open peer review process please email info.bmjopen@bmj.com

BMJ Open

Self-rated health and physical activity during retirement transition – a multi-trajectory analysis of concurrent changes

Journal:	*BMJ Open*
Manuscript ID	bmjopen-2023-073876
Article Type:	Original research
Date Submitted by the Author:	21-Mar-2023
Complete List of Authors:	Lintuaho, Roosa; TYKS Turku University Hospital, Department of Emergency Medicine; TYKS Turku University Hospital, Department of Physical and Rehabilitation Medicine<br>Saltychev, Mikhail; TYKS Turku University Hospital, Department of Physical and Rehabilitation Medicine<br>Pentti, Jaana; University of Turku, Department of Public Health; University of Helsinki Clinicum Unit<br>Vahtera, Jussi; University of Turku, Department of Public Health; University of Turku, Centre for Population Health Research<br>Stenholm, Sari; University of Turku, Department of Public Health; University of Turku, Centre for Population Health Research
Keywords:	PUBLIC HEALTH, Aging, Life Change Events

SCHOLARONE™
Manuscripts

I, the Submitting Author has the right to grant and does grant on behalf of all authors of the Work (as defined in the below author licence), an exclusive licence and/or a non-exclusive licence for contributions from authors who are: i) UK Crown employees; ii) where BMJ has agreed a CC-BY licence shall apply, and/or iii) in accordance with the terms applicable for US Federal Government officers or employees acting as part of their official duties; on a worldwide, perpetual, irrevocable, royalty-free basis to BMJ Publishing Group Ltd ("BMJ") its licensees and where the relevant Journal is co-owned by BMJ to the co-owners of the Journal, to publish the Work in this journal and any other BMJ products and to exploit all rights, as set out in our licence.

The Submitting Author accepts and understands that any supply made under these terms is made by BMJ to the Submitting Author unless you are acting as an employee on behalf of your employer or a postgraduate student of an affiliated institution which is paying any applicable article publishing charge ("APC") for Open Access articles. Where the Submitting Author wishes to make the Work available on an Open Access basis (and intends to pay the relevant APC), the terms of reuse of such Open Access shall be governed by a Creative Commons licence – details of these licences and which Creative Commons licence will apply to this Work are set out in our licence referred to above.

Other than as permitted in any relevant BMJ Author's Self Archiving Policies, I confirm this Work has not been accepted for publication elsewhere, is not being considered for publication elsewhere and does not duplicate material already published. I confirm all authors consent to publication of this Work and authorise the granting of this licence.

For peer review only

Self-rated health and physical activity during retirement transition – a multi-trajectory analysis of concurrent changes

Roosa Lintuaho[1,2], Mikhail Saltychev[2], Jaana Pentti[3,4,5], Jussi Vahtera[3,4], Sari Stenholm[3,4]

[1] Department of Emergency Medicine, Turku University Hospital and University of Turku, Finland

[2] Department of Physical and Rehabilitation Medicine, Turku University Hospital and University of Turku, Turku, Finland

[3] Department of Public Health, University of Turku and Turku University Hospital, Turku, Finland

[4] Centre for Population Health Research, University of Turku and Turku University Hospital; Turku, Finland

[5] Clinicum, Faculty of Medicine, University of Helsinki, Helsinki, Finland

Corresponding author: Roosa Lintuaho, roemti@utu.fi

For peer review only

DECLARATIONS

Ethics approval and consent to participate: A written informed consent was obtained from all the participants. The study was approved by the Ethics Committee of Hospital District of Southwest Finland.

Consent for publication: Not applicable

Availability of data and material: Anonymised partial datasets of the Finnish Retirement and Aging Study are available by application with bona fide researchers with an established scientific record and bona fide organisations. For more information, please contact Professor Sari Stenholm (firea@utu.fi).

Competing interests: None

Funding: This study was supported by funding granted by the Academy of Finland (321409 and 329240 to JV, 286294, 319246, 294154, 332030 to SS), Finnish Ministry of Education and Culture (to SS); Juho Vainio Foundation (to SS), and Competitive State Research Financing of the Expert Responsibility Area of the Turku University Hospital (to RL and SS)

Author statement: All the authors (RL, MS, JP, JV, SS) substantially contributed to the conception and design of the work, the interpretation of the results and revising it critically for important intellectual content. SS and JV were responsible for the acquisition of data for the work. MS and JP were responsible for the statistical analysis. RL was responsible for drafting the work. All the authors (RL, MS, JP, JV, SS) have finally approved the version to be published and they are agreed to be accountable for all aspects of the work in ensuring that questions related to the accuracy or integrity of any part of the work are appropriately investigated and resolved.

Acknowledgements: None

For peer review only

Word count: 2939

For peer review only

For peer review only - http://bmjopen.bmj.com/site/about/guidelines.xhtml

ABSTRACT

Objectives: The aim of the study was to evaluate concurrent changes in physical activity and self-rated health during retirement transition by multivariate trajectory analysis and to examine, whether sociodemographic and lifestyle factors predict the probability of being classified to a certain subgroup of observed changes.

Design: prospective cohort study

Setting: public sector employees

Participants: 3,550 participants of the Finnish Retirement and Aging (FIREA) study

Primary and secondary outcome measures: Participants estimated on a yearly questionnaire their weekly hours of different types of activities converted to MET-h/week. Self-rated health was assessed on a 5-point Likert-like scale from poor to excellent and dichotomized as suboptimal and optimal. Multivariate trajectory analysis was used to distinguish different subgroups of trajectories. Multinomial regression analysis was used to describe the associations between covariates and the probability of being classified to a certain trajectory group.

Results: Three trajectory groups were identified, all displaying increasing activity during retirement with a simultaneous decrease in perceived suboptimal health. The improvement was temporary for most, except for 9% of participants who had high physical activity which increased during follow-up, with low percentage of suboptimal health. Male gender, professional occupation, being married or co-habiting, normal or overweight, not smoking and using alcohol below risk levels were associated with higher activity and better self-rated health.

Conclusion: Changes in physical activity and perceived health during retirement transition were interconnected. Both were improved during retirement transition, but the change was temporary. Longer follow-up studies are required to assess the changes over longer period after retirement.

Keywords: retirement; physical activity; self-rated health

For peer review only

Strengths and limitations of this study

- A data-driven approach to finding subgroups with different trajectory paths.
- Large sample size, representative of the public sector workforce.
- Repeated measurements of self-rated health and physical activity.
- The self-reported nature of the variables might lead to information bias.
- Participants were mostly retiring due to old age, leaving out those retiring due to health reasons, who might get different results.

For peer review only

For peer review only - http://bmjopen.bmj.com/site/about/guidelines.xhtml

INTRODUCTION

Retirement may change many daily routines, such as e.g., diet, sleeping rhythm, psychological well-being, as well as the types and intensity of leisure-time activities or hobbies.[1-4] Such a comprehensive change may also affect health perception.

Several studies have reported increasing physical activity during the retirement transition.[1 5-12] According to multiple follow-up studies, this increase might be temporary.[6 10 13] The type of retirement affects the change in physical activity – retiring due to health reasons has been associated with lesser increase or even decreasing physical activity after retirement.[7 9] Gender, work characteristics and socioeconomic status may also moderate these changes. Studies by Touvier et al.[14] and Lahti et al.[7] have reported greater increase in physical activity during retirement transition among men, whereas study by Sjösten et al.[5] has showed greater increase in physical activity among retiring women. Retiring from non-manual work seems to be beneficial regarding physical activity comparing with retiring from manual jobs.[6 15 16] Systematic reviews have also reported the effect of socioeconomic status on the intensity of physical activity after retirement – higher status has been associated with improved physical activity.[1 17] Some sex-related differences have been reported concerning the effect of occupational status – women retiring from manual work have showed decreased accelerometer-measured physical activity, while physical activity has not changed among women retiring from non-manual work.[16] Respectively, men retiring from non-manual work had increased physical activity, while those retiring from manual work have not demonstrated any change.[16]

Previous knowledge on the change in self-rated health during retirement transition has been inconsistent. In a French study among employees, who retired at relatively young age (54 years on average), perceived health has improved after retirement, especially for those with poorer working conditions and health problems prior to retirement.[18] In a systematic review, mental health has

For peer review only - http://bmjopen.bmj.com/site/about/guidelines.xhtml

often substantially improved after retirement. Instead, the change in self-rated health has been less clear as different studies have reported improvement, deterioration or no change in self-rated health.[19] A meta-analysis by van der Heide et al. has reported either deterioration or improvement in self-rated health depending on which set of original studies has been pooled.[19] It seems that retirement due to health reasons is associated with greater positive changes in perceived health compared with those retired due to old age.[19] Worsening physical health has been associated with lower occupational status, manual work and higher prevalence of chronic conditions.[20] In another study, job strain and physically demanding work have been associated with suboptimal health prior to retirement. In turn, sustained good health has been associated with normal weight, non-smoking and higher physical activity.[21]

Previous research has found associations between physical activity and self-rated health.[21 22] Thus, it can be assumed that changes in physical activity and self-rated health may follow the same patterns around the retirement transition. Simultaneous changes on the subject have not been studied before.

The aim of this study was to evaluate concurrent changes in physical activity and self-rated health during retirement transition by using a multi-trajectory analysis to identify subgroups with different trajectory paths during this transition.

For peer review only - http://bmjopen.bmj.com/site/about/guidelines.xhtml

METHODS

Study population

The Finnish Retirement and Aging Study (FIREA) is an ongoing longitudinal cohort study, which follows aging workers from final years in working life to full-time retirement and until old age.[23] The eligible population for the FIREA included all public sector employees whose individual retirement date was between 2014 and 2019 and who were working in the year 2012 in one of 27 municipalities, nine cities or five hospital districts included in the study.

The participants were first contacted 18 months prior to their estimated retirement date, which was obtained from the register kept by the pension provider for public sector employees (Keva). The actual retirement date was self-reported. The participants received a questionnaire, which has been thereafter sent annually, at least four times. Participants who had provided information regarding physical activity and self-rated health at least two consecutive times, one right before retirement and one after, were included in the current study (n=3,550). There were two possible survey waves before the retirement, waves -2 and -1, and three possible waves after the retirement, waves 1, 2 and 3 (Figure 1).

All the respondents have provided a written informed consent. The FIREA is following the Declaration of Helsinki and has been approved by the Ethics Committee of Hospital District of Southwest Finland.

Measurement of physical activity and self-rated health

Physical activity was self-reported at each study wave. The participants were asked to estimate their average weekly hours of leisure-time physical activity (including commuting) in activities comparable to walking, brisk walking, jogging or running..[24] The level of physical activity was then

For peer review only - http://bmjopen.bmj.com/site/about/guidelines.xhtml

converted into metabolic equivalent of task (MET), which describes the amount of consumed energy comparing to resting. One MET unit of 3.5 ml/kg/minute corresponds to oxygen consumption while sitting at rest. Weekly physical activity was expressed as MET-h/week. To help the interpretation of the results, following categorization was used: low (<14 MET-h/week), moderate (14 to <30 MET-h/week) or high (≥30 MET-h/week) physical activity.[24 25] This categorization was chosen since physical activity lower than 14 MET-h/week (approximately the equivalent of 140 minutes of brisk walking) has been reported to be associated with higher risk of cardiovascular disease[26] and the activity level of 30 MET-h/week (the equivalent of 300 minutes of brisk walking) has been shown to be needed for weight management.[27]

Self-reported health was inquired at each study wave. Participants were asked to rate their health on a five-point Likert-like scale:1-good, 2-rather good, 3-average, 4-rather poor or 5-poor. For the purpose of analysis, the result was dichotomized as 3-5 as "suboptimal health" vs. 1-2 as "optimal health".

Independent variables

Age was defined in years. Marital status was dichotomized as "married or co-habiting" vs. "single". Occupational titles were obtained from the register of pension provider (Keva), and they were coded according to the International Standard Classification of Occupations (ISCO) and dichotomized as "professionals" (ISCO major groups 1 – 4) vs. "service and manual workers" (ISCO major groups 5 – 9). Body Mass Index (BMI) was calculated as weight/height^2 according to self-reported body height and weight. BMI was dichotomized as "normal and overweight" (BMI < 30kg/m^2) vs. "obesity" (≥30kg/m^2). Smoking was dichotomized as "current smokers" vs. "never-smokers and former smokers". Alcohol consumption was obtained from the survey as units consumed weekly and

For peer review only - http://bmjopen.bmj.com/site/about/guidelines.xhtml

converted into grams of pure alcohol per week (g/week). Amount of >288 g/week for men and >192 g/week for women was considered a cut-off for excess alcohol consumption and dichotomized as "risk-users "vs. "non-risk-users". Information on covariates were obtained at the last available wave before retirement, wave -1.

Statistical analysis

The covariates were reported as means and standard deviations or as absolute numbers and percentage, when appropriate.

Group-based multi-trajectory analysis (GBTA) was used to investigate the developmental trajectories (a course of outcome over time) of physical activity and self-reported health. The group-based multi-trajectory analysis is a form of finite mixture modelling for analysing longitudinal repeated measures data.[28] It is a data-driven form of analysis which is able to distinguish and describe subpopulations (clusters) existing within a studied population, while conventional statistics only show the average outcome over time. The trajectories of such subpopulations may differ substantially from each other and from the average trajectory observed in the entire population. The censored (known also as 'regular') normal model of group-based multi-trajectory analysis was used. The goodness of model fit was judged by running the procedure several times with a number of clusters starting from one up to four. The Bayesian Information Criterion (BIC), Akaike information criterion (AIC) and average posterior probability (APP) were used as criteria to confirm the goodness of fit. Linear, quadratic and cubic regression models were tested and cubic model was retained for using in the analysis. The cut-off for the smallest group was set at ≥5% of the entire cohort. Physical activity was treated as a continuous variable. Self-rated health

For peer review only

was dichotomized in the analysis as suboptimal (yes/no). For sensitivity analysis, self-rated health was also used as a continuous variable.

Multinomial regression analysis was used to describe the associations of demographic and lifestyle factors and probability of being classified into a particular cluster. The results were presented as risk ratios (RRs) and their 95% confidence intervals (95% Cis). The RRs were adjusted for age and gender (only for age for gender RR).

The analyses were performed using Stata/IC Statistical Software: Release 17. College Station (StataCorp LP, TX, USA). The additional Stata module 'traj' was required to conduct group-based trajectory analysis. The module is freely available for both SAS® and Stata software.[29]

For peer review only

RESULTS

The characteristics of the study sample prior to retirement (study wave -1) are presented in Table 1. Of the respondents, 83% were women, 31% were single and 65% were professional workers. Mean age before the retirement was 63.4 (SD 1.4) years. Only 9% were currently smoking, 2% consumed alcohol over risk limits and 28% had a BMI >=30kg/m^2. Of the respondents, 24% rated their health as suboptimal and 22% reported low, 18% moderate and 60% high physical activity.

For peer review only

Trajectory groups

Three trajectories of concurrent changes in physical activity and self-rated health were identified (Figure 2). The three-cluster model was chosen, since the smallest group in the next, four-cluster model, fell below the pre-defined cut-off of 5% (Table 2). The smallest APP was sufficient 0.93 for the three-cluster model. Compared to the two-cluster model, a three-cluster model demonstrated BIC and AIC values closer to zero. The results are also presented using the same scale for a Y-axis in Supplement 1. The estimates of physical activity and self-rated health by trajectory group at each wave are presented in Supplement 2.

Group #1: Moderate physical activity and suboptimal self-rated health (23%)

Physical activity was at a moderate level of 17.1 MET-h/week six months before retirement. First, it slightly increased from final working years until 18 months after retirement up to 18.1 MET-h/week and thereafter slightly decreased. The trajectory of self-rated health was mirroring the changes in physical activity: 79% reported suboptimal health six months prior to retirement improving down to 71% 18 months after the retirement and then slightly worsening again.

Group #2: Moderate physical activity and good self-rated health (69%)

Physical activity was at a moderate level of 22.6 MET-h/week six months before retirement and increased during retirement transition all the way to 18 months after the retirement to 24.4. MET-

For peer review only - http://bmjopen.bmj.com/site/about/guidelines.xhtml

h/week. After that, physical activity showed a mild decrease staying, however, higher than the initial level. In this group, only 6% of the respondent reported suboptimal health six months before the retirement and decreasing down to 3% on the way to 18 months after the retirement. After that time point, the percentage of people with suboptimal health grew again up to 6%.

Group #3: High physical activity and good self-rated health (9%)

This smallest group demonstrated improvement in both studied parameters during the entire follow-up. The intensity of physical activity increased from 62.6 MET-h/week six months before retirement up to 65 MET-h/week 18 months after retirement. Respectively, the portion of respondents with suboptimal health decreased from 7% down to 4%.

Sensitivity analysis

The trajectory solution was similar when the analysis was rerun treating self-rated health as a continuous variable instead of binary variable (Supplement 3). The respective groups were:

Group #1: moderate physical activity and suboptimal self-rated health (31%)

Group #2: moderate physical activity and good self-rated health (61%)

Group #3: increasing high physical activity and good self-rated health (8%).

Associations between sociodemographic and health behavioural factors and trajectory groups

Compared to group #1 (moderate physical activity and suboptimal self-rated health), in groups #2(moderate physical activity and good self-rated health) and #3 (increasing physical activity and improving self-rated health), there were more married or co-habiting persons (RR 1.23, 95%CI 1.04-1.47/RR 1.48, 95% CI 1.10-1.99), less manual workers (RR 0.77, 95%CI 0.65-0.91/RR 0.66, 95% CI 0.50-0.88), less overweight or obese participants (RR 0.35, 95%CI 0.30-042/RR 0.13, 95% CI 0.09-

For peer review only - http://bmjopen.bmj.com/site/about/guidelines.xhtml
For peer review only

0.19), less smokers (RR 0.68, 95%CI 0.52-0.88/RR 0.31, 95% CI 0.17-0.58) and less risk-users of alcohol (RR 0.60, 95% CI 0.38-0.96/RR 0.23, 95% CI 0.07-0.77). (Table 3)

When comparing the most active and healthy groups #2 and #3, there were less women (RR 0.63, 95% CI 0.47-0.84), less persons with BMI >30 kg/m^2 (RR 0.36, 95% CI 0.25-0.53) and smokers (RR 0.46, 95% CI 0.25-0.83) in group #3 than in group #2 (Table 3).

For peer review only

For peer review only - http://bmjopen.bmj.com/site/about/guidelines.xhtml

Discussion

In this prospective cohort study among 3,550 public sector employees, three trajectory groups of concurrent changes in physical activity and self-rated health were identified. Overall, in all three groups, physical activity and self-rated health were clearly interconnected; trajectories for both parameters were mirroring each other – increase in physical activity was accompanied by improving health perception and vice versa. Higher physical activity was clearly associated with better self-rated health. These findings mirror previous reports on association between higher physical activity and better self-rated health.[22 30 31] However, concurrent trajectories of changes in these two parameters have not been studied before.

Overall, physical activity and self-rated health increased simultaneously during retirement transition. For 91% of the participants, this increase was only temporary. In regards of physical activity, this has been seen in previous research as well[6 10 13]. Only in a small proportion of participants, who were initially highly active and in good health, the improvement was maintained after the retirement transition throughout the entire follow-up. In this group, there were more men, professional workers and participants with BMI <30 kg/m^2 than in other groups, which is in line with previous findings; higher physical activity after retirement has been associated with higher socioeconomical status[1 12 17], normal weight[12] and in some studies, male sex[7 14].

Some previous studies have observed different paths of self-rated health. A study from New Zealand, identified three trajectories of self-rated health: declining, recovering and one with rather stable self-rated health that has showed only a minor decrease after retirement.[20] In a Finnish study, four trajectories around retirement transition were observed: two with sustained level of health, either good or suboptimal, one with improving self-rated health and one with declining health.[21] The difference in these results compared to current could be due to the difference in study samples,

For peer review only - http://bmjopen.bmj.com/site/about/guidelines.xhtml

but also due to the multi-trajectory-setting of the current study, in which changes in physical activity are just as important when defining the trajectories.

Compared to the group with moderate activity and poor self-rated health, there were more professional workers, persons with normal weight, non-smokers, co-habiting participants and participants who consume alcohol within moderation in the other two groups with higher activity and better self-rated health. These findings are in line with previous work; higher physical activity after retirement has been associated with retiring form professional work and higher socioeconomical status[1 6 17] and lower BMI[12]. Poorer self-rated health has also been found to be associated with lower socioeconomic status, overweight and lower physical activity,[21 32] and good self-rated health has been associated with normal weight, non-smoking and normal level of physical activity.[21]

Although there was improvement in all groups regarding physical activity and self-rated health, the improvement in self-rated health was most prominent in the group with lowest activity and poorer self-rated health; the proportion of participants with suboptimal health decreased by 12%, whereas in other groups the change was 3 to 6% during follow-up (although due to small percentage of individuals experiencing suboptimal health in these groups, such an improvement wasn't possible). In this group, there was more single people, manual workers, participants with obesity, current smokers, and participants with risk-use of alcohol. This has been seen in other studies as well; in the New Zealandean study, retirement has been most beneficial in regards of self-rated health to those with manual occupations, low to medium socioeconomic statuses and multiple chronic conditions.[20] On the other hand, some studies have found retirement to be associated with declining self-rated health for people with higher socioeconomic status, which was not the case in the current study.[19 20]

For peer review only - http://bmjopen.bmj.com/site/about/guidelines.xhtml

Strengths and limitations

This study was conducted on a large cohort, representative of the public sector workforce. Strengths of this study also include a data-driven approach to defining clusters and repeated measurements of physical activity and self-rated health.

There are some limitations as well, which should be considered. The participants were, due to the inclusion criteria, mostly retiring due to old age. Since most of the participants could keep on working until their retirement age, they were probably healthier and in better shape than those who retired earlier due to health reasons. Although the sample is representative of public sector employees in Finland, the sample was predominated by women (83%) and professional workers (65%), which affects the generalisability of these findings into general population. This study only assessed leisure-time and commuting physical activity, leaving out work-related activity. The self-reported nature of the estimates could lead to information bias. Self-rated health is, although, a widely used measurement perceived as a valid indicator of sustaining well-being. It is an independent predictor of mortality[33], as well as increased use of prescribed medications and healthcare and social services[34]. To minimize the risk of bias due to the measurement used, the analyses were conducted separately with self-rated health treated as a dichotomized variable and as a continuous variable.

Further research is needed on interventions on how to maintain the positive trend in self-rated health and physical activity in the years following retirement.

Conclusions

Physical activity was found to increase, and self-rated health improve during retirement transition, although this change was temporary for most participants. Changes in physical activity and self-rated health seemed to be interconnected. Lower physical activity, being single, manual work,

For peer review only

obesity, current smoking, and risk-use of alcohol were associated with poorer self-rated health and lower physical activity.

For peer review only

For peer review only - http://bmjopen.bmj.com/site/about/guidelines.xhtml

References

1. Barnett I, van Sluijs EM, Ogilvie D. Physical activity and transitioning to retirement: a systematic review. *Am J Prev Med* 2012;43(3):329-36. doi: 10.1016/j.amepre.2012.05.026 [published Online First: 2012/08/18]
2. Ali-Kovero K, Pietilainen O, Mauramo E, et al. Changes in fruit, vegetable and fish consumption after statutory retirement: a prospective cohort study. *Br J Nutr* 2020;123(12):1390-95. doi: 10.1017/S0007114520000136 [published Online First: 20200120]
3. Vahtera J, Westerlund H, Hall M, et al. Effect of retirement on sleep disturbances: the GAZEL prospective cohort study. *Sleep* 2009;32(11):1459-66. doi: 10.1093/sleep/32.11.1459
4. Lahdenpera M, Virtanen M, Myllyntausta S, et al. Psychological Distress During the Retirement Transition and the Role of Psychosocial Working Conditions and Social Living Environment. *J Gerontol B Psychol Sci Soc Sci* 2022;77(1):135-48. doi: 10.1093/geronb/gbab054
5. Sjosten N, Kivimaki M, Singh-Manoux A, et al. Change in physical activity and weight in relation to retirement: the French GAZEL Cohort Study. *BMJ Open* 2012;2:e000522. doi: 10.1136/bmjopen-2011-000522 [published Online First: 2012/02/10]
6. Stenholm S, Pulakka A, Kawachi I, et al. Changes in physical activity during transition to retirement: a cohort study. *Int J Behav Nutr Phys Act* 2016;13:51. doi: 10.1186/s12966-016-0375-9 [published Online First: 2016/04/17]
7. Lahti J, Holstila A, Manty M, et al. Changes in leisure time physical activity and subsequent disability retirement: A register-linked cohort study. *Int J Behav Nutr Phys Act* 2016;13(1):99. doi: 10.1186/s12966-016-0426-2 [published Online First: 20160906]
8. Engberg E, Alen M, Kukkonen-Harjula K, et al. Life events and change in leisure time physical activity: a systematic review. *Sports Med* 2012;42(5):433-47. doi: 10.2165/11597610-000000000-00000
9. Feng X, Croteau K, Kolt GS, et al. Does retirement mean more physical activity? A longitudinal study. *BMC Public Health* 2016;16:605. doi: 10.1186/s12889-016-3253-0 [published Online First: 20160720]
10. Holstila A, Manty M, Rahkonen O, et al. Statutory retirement and changes in self-reported leisure-time physical activity: a follow-up study with three time-points. *BMC Public Health* 2017;17(1):528. doi: 10.1186/s12889-017-4455-9 [published Online First: 20170530]
11. Henning G, Stenling A, Bielak AAM, et al. Towards an active and happy retirement? Changes in leisure activity and depressive symptoms during the retirement transition. *Aging Ment Health* 2021;25(4):621-31. doi: 10.1080/13607863.2019.1709156 [published Online First: 20200122]
12. Lintuaho R, Saltychev M, Pentti J, et al. Multi-trajectory analysis of changes in physical activity and body mass index in relation to retirement: Finnish Retirement and Aging study. *PLoS One* 2022;17(12):e0278405. doi: 10.1371/journal.pone.0278405 [published Online First: 20221201]
13. Van Dyck D, Cardon G, De Bourdeaudhuij I. Longitudinal changes in physical activity and sedentary time in adults around retirement age: what is the moderating role of retirement status, gender and educational level? *BMC Public Health* 2016;16(1):1125. doi: 10.1186/s12889-016-3792-4 [published Online First: 20161028]
14. Touvier M, Bertrais S, Charreire H, et al. Changes in leisure-time physical activity and sedentary behaviour at retirement: a prospective study in middle-aged French subjects. *Int J Behav Nutr Phys Act* 2010;7:14. doi: 10.1186/1479-5868-7-14 [published Online First: 20100204]

For peer review only - http://bmjopen.bmj.com/site/about/guidelines.xhtml

15. Chung S, Domino ME, Stearns SC, et al. Retirement and physical activity: analyses by occupation and wealth. *Am J Prev Med* 2009;36(5):422-8. doi: 10.1016/j.amepre.2009.01.026 [published Online First: 20090306]
16. Pulakka A, Leskinen T, Suorsa K, et al. Physical Activity across Retirement Transition by Occupation and Mode of Commute. *Med Sci Sports Exerc* 2020;52(9):1900-07. doi: 10.1249/MSS.0000000000002326 [published Online First: 2020/03/10]
17. Vansweevelt N, Boen F, van Uffelen J, et al. Socioeconomic Differences in Physical Activity and Sedentary Behavior During the Retirement Transition: A Systematic Review of Longitudinal Studies. *J Phys Act Health* 2022:1-15. doi: 10.1123/jpah.2022-0196 [published Online First: 20220819]
18. Westerlund H, Kivimaki M, Singh-Manoux A, et al. Self-rated health before and after retirement in France (GAZEL): a cohort study. *Lancet* 2009;374(9705):1889-96. doi: 10.1016/S0140-6736(09)61570-1 [published Online First: 20091110]
19. van der Heide I, van Rijn RM, Robroek SJ, et al. Is retirement good for your health? A systematic review of longitudinal studies. *BMC Public Health* 2013;13:1180. doi: 10.1186/1471-2458-13-1180 [published Online First: 20131213]
20. Szabo A, Allen J, Stephens C, et al. Is retirement associated with physical health benefits? A longitudinal investigation with older New Zealanders. *Age Ageing* 2019;48(2):267-72. doi: 10.1093/ageing/afy176
21. Stenholm S, Virtanen M, Pentti J, et al. Trajectories of self-rated health before and after retirement: evidence from two cohort studies. *Occup Environ Med* 2020;77(2):70-76. doi: 10.1136/oemed-2019-106026 [published Online First: 20191211]
22. Sperlich S, Beller J, Epping J, et al. Trends in self-rated health among the elderly population in Germany from 1995 to 2015 - the influence of temporal change in leisure time physical activity. *BMC Public Health* 2020;20(1):113. doi: 10.1186/s12889-020-8218-7 [published Online First: 20200128]
23. Leskinen T, Pulakka A, Heinonen OJ, et al. Changes in non-occupational sedentary behaviours across the retirement transition: the Finnish Retirement and Aging (FIREA) study. *J Epidemiol Community Health* 2018;72(8):695-701. doi: 10.1136/jech-2017-209958 [published Online First: 20180410]
24. Kujala UM, Kaprio J, Sarna S, et al. Relationship of leisure-time physical activity and mortality: the Finnish twin cohort. *JAMA* 1998;279(6):440-4. doi: 10.1001/jama.279.6.440 [published Online First: 1998/02/18]
25. Leskinen T, Stenholm S, Heinonen OJ, et al. Change in physical activity and accumulation of cardiometabolic risk factors. *Prev Med* 2018;112:31-37. doi: 10.1016/j.ypmed.2018.03.020 [published Online First: 2018/04/02]
26. Tanasescu M, Leitzmann MF, Rimm EB, et al. Exercise type and intensity in relation to coronary heart disease in men. *JAMA* 2002;288(16):1994-2000. doi: 10.1001/jama.288.16.1994 [published Online First: 2002/10/22]
27. Fogelholm M, Suni, J., Rinne, M., Oja, P., Vuori, I. Physical Activity Pie: A Graphical Presentation Integrating Recommendations for Fitness and Health. *Journal of Physical Activity and Health* 2005;2:391-96.
28. Nagin DS, Odgers CL. Group-Based Trajectory Modeling (Nearly) Two Decades Later. *J Quant Criminol* 2010;26(4):445-53. doi: 10.1007/s10940-010-9113-7 [published Online First: 2010/12/07]
29. Jones BL ND. Traj - Group-based modeling of longitudinal data 1999 [updated 9/24/1999.

For peer review only

30. Park S, Lee JH. Associations of occupational and leisure-time physical activity with self-rated health in Korea. *Prev Med* 2022;158:107022. doi: 10.1016/j.ypmed.2022.107022 [published Online First: 20220317]
31. Papp ME, Grahn-Kronhed AC, Rauch Lundin H, et al. Changes in physical activity levels and relationship to balance performance, gait speed, and self-rated health in older Swedish women: a longitudinal study. *Aging Clin Exp Res* 2022;34(4):775-83. doi: 10.1007/s40520-021-02016-5 [published Online First: 20211116]
32. Hoertel N, Rico MS, Limosin F, et al. Retirement age does not modify the association of prior working conditions with self-rated health and mortality in retirees: results from a prospective study of retired French workers. *Int Arch Occup Environ Health* 2022 doi: 10.1007/s00420-022-01886-0 [published Online First: 20220610]
33. Jylha M. What is self-rated health and why does it predict mortality? Towards a unified conceptual model. *Soc Sci Med* 2009;69(3):307-16. doi: 10.1016/j.socscimed.2009.05.013 [published Online First: 20090610]
34. Bath PA. Self-rated health as a risk factor for prescribed drug use and future health and social service use in older people. *J Gerontol A Biol Sci Med Sci* 1999;54(11):M565-70. doi: 10.1093/gerona/54.11.m565

For peer review only

Table 1. Pre-retirement characteristics of the study population (wave -1)

Variable	n	%
Gender		
Women	2,949	83
Men	601	17
Marital status		
Single	1,088	31
Co-habiting	2,462	69
ISCO class		
Professional	2,283	65
Manual or service worker	1,239	35
BMI		
BMI <30 kg/m^2	2,569	72
BMI >=30 kg/m^2	981	28
Smoking		
Not currently	3,192	91
Currently smoking	305	8.72
Alcohol consumption		
No risk-use	3,457	98
Risk-use	87	2
Suboptimal self-rated health		
No	2,690	76
Yes	853	24
Physical activity		
Low (<14 MET-h/week)	770	22
Moderate (14 to <30 MET-h/week)	638	18
High (≥30 MET-h/week)	2,142	60
	Mean	SD
Age	63.4	1.4

For peer review only

Table 2. Goodness of fit of group-based trajectory analysis models. The chosen model is shown in bold. Self-rated health treated as a binary variable.

Number of clusters	Shape of trajectory	Smallest group		BIC[1]	AIC[2]	Smallest APP[3]
		N	%			
1	Cubic	3,550	100	66,605	66,568	1.0
2	Cubic	861	24	64,626	64,552	0.94
**3**	**Cubic**	**308**	**9**	**62,956**	**62,845**	**0.93**
4	Cubic	64	2	62,434	62,286	0.89

[1] BIC = Bayesian Information Criterion, [2] AIC = Akaike information criterion, [3] APP = Smallest average posterior probability

For peer review only

Table 3. Risk ratios (RR) and their 95% confidence intervals (CI:s) of being classified to a certain trajectory group. RRs are adjusted for age and gender (only for age for gender RR). Statistically significant findings are shown in bold.

	Group #1			Group #2			Group #3		
**Variable/group**	**RR**	**95% CI**		**RR**	**95% CI**		**RR**	**95% CI**	
**Women vs. men**									
Group #1: Moderate physical activity and suboptimal self-rated health	1			0.82	0.66	1.01	1.29	0.94	1.78
Group #2: Moderate physical activity and good self-rated health	1.23	0.99	1.51	1			**1.59**	**1.19**	**2.11**
Group #3: increasing physical activity and improving self-rated health	0.77	0.56	1.07	**0.63**	**0.47**	**0.84**	1		
**Co-habiting vs. single**									
Group #1: Moderate physical activity and suboptimal self-rated health	1			**0.81**	**0.68**	**0.96**	**0.68**	**0.50**	**0.91**
Group #2: Moderate physical activity and good self-rated health	**1.23**	**1.04**	**1.47**	1			0.84	0.64	1.10
Group #3: increasing physical activity and improving self-rated health	**1.48**	**1.10**	**1.99**	1.20	0.91	1.57	1		
**Manual workers vs. professional workers**									
Group #1: Moderate physical activity and suboptimal self-rated health	1			**1.30**	**1.10**	**1.53**	**1.52**	**1.14**	**2.02**
Group #2: Moderate physical activity and good self-rated health	**0.77**	**0.65**	**0.91**	1			1.17	0.90	1.52
Group #3: increasing physical activity and improving self-rated health	**0.66**	**0.50**	**0.88**	0.86	0.66	1.11	1		
**BMI ≥30 vs. BMI <30**									
Group #1: Moderate physical activity and suboptimal self-rated health	1			**2.82**	**2.38**	**3.33**	**4.05**	**5.23**	**11.58**
Group #2: Moderate physical activity and good self-rated health	**0.35**	**0.30**	**0.42**	1			**2.76**	**1.88**	**4.05**
Group #3: increasing physical activity and improving self-rated health	**0.13**	**0.09**	**0.19**	**0.36**	**0.25**	**0.53**	1		
**Current smokers vs. former/never smokers**									
Group #1: Moderate physical activity and suboptimal self-rated health	1			**1.47**	**1.13**	**1.90**	**3.19**	**1.72**	**5.93**
Group #2: Moderate physical activity and good self-rated health	**0.68**	**0.52**	**0.88**	1			**2.18**	**1.20**	**3.95**
Group #3: increasing physical activity and improving self-rated health	**0.31**	**0.17**	**0.58**	**0.46**	**0.25**	**0.83**	1		
**Risk-users of alcohol vs. non-risk users**									
Group #1: Moderate physical activity and suboptimal self-rated health	1			**1.66**	**1.05**	**2.62**	**4.33**	**1.31**	**14.39**
Group #2: Moderate physical activity and good self-rated health	**0.60**	**0.38**	**0.96**	1			2.62	0.81	8.46
Group #3: increasing physical activity and improving self-rated health	**0.23**	**0.07**	**0.77**	0.38	0.12	1.24	1		

For peer review only

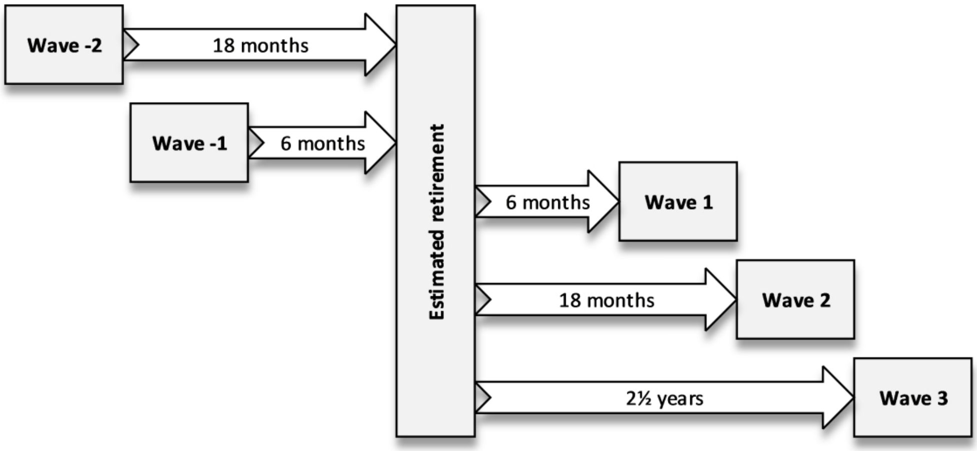

Figure 1. Study waves and time in relation to retirement

639x297mm (120 x 120 DPI)

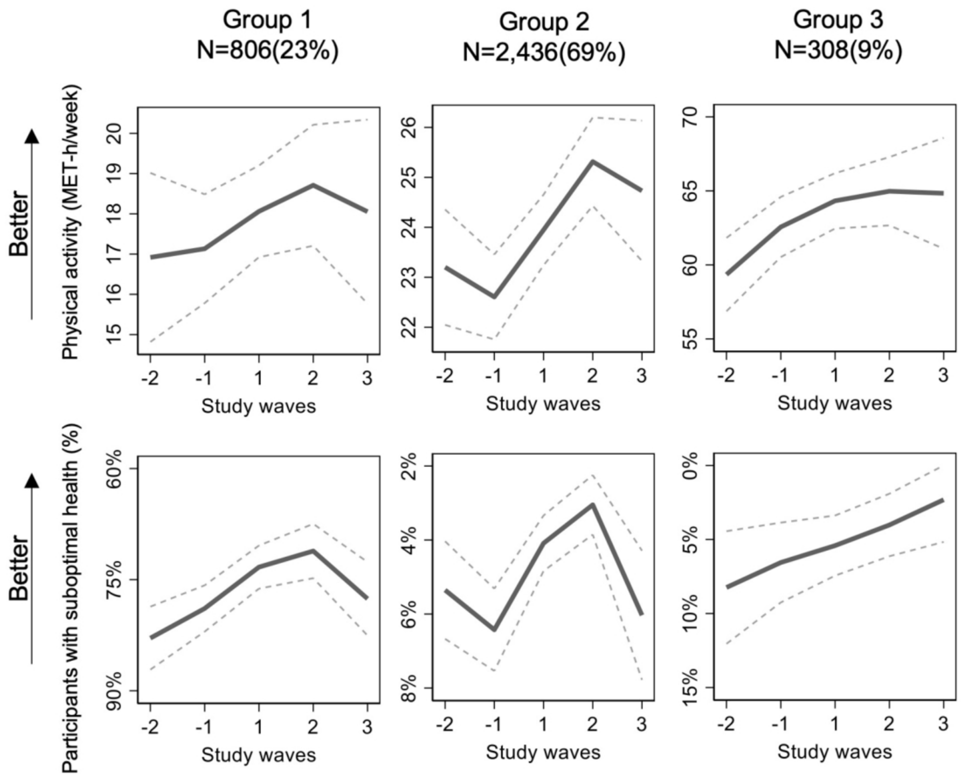

Figure 2. Trajectories of physical activity and self-rated health. The scale is different in each group.

139x117mm (300 x 300 DPI)

Supplement 1. Trajectories of physical activity and self-rated health. All the groups are presented within the same scale.

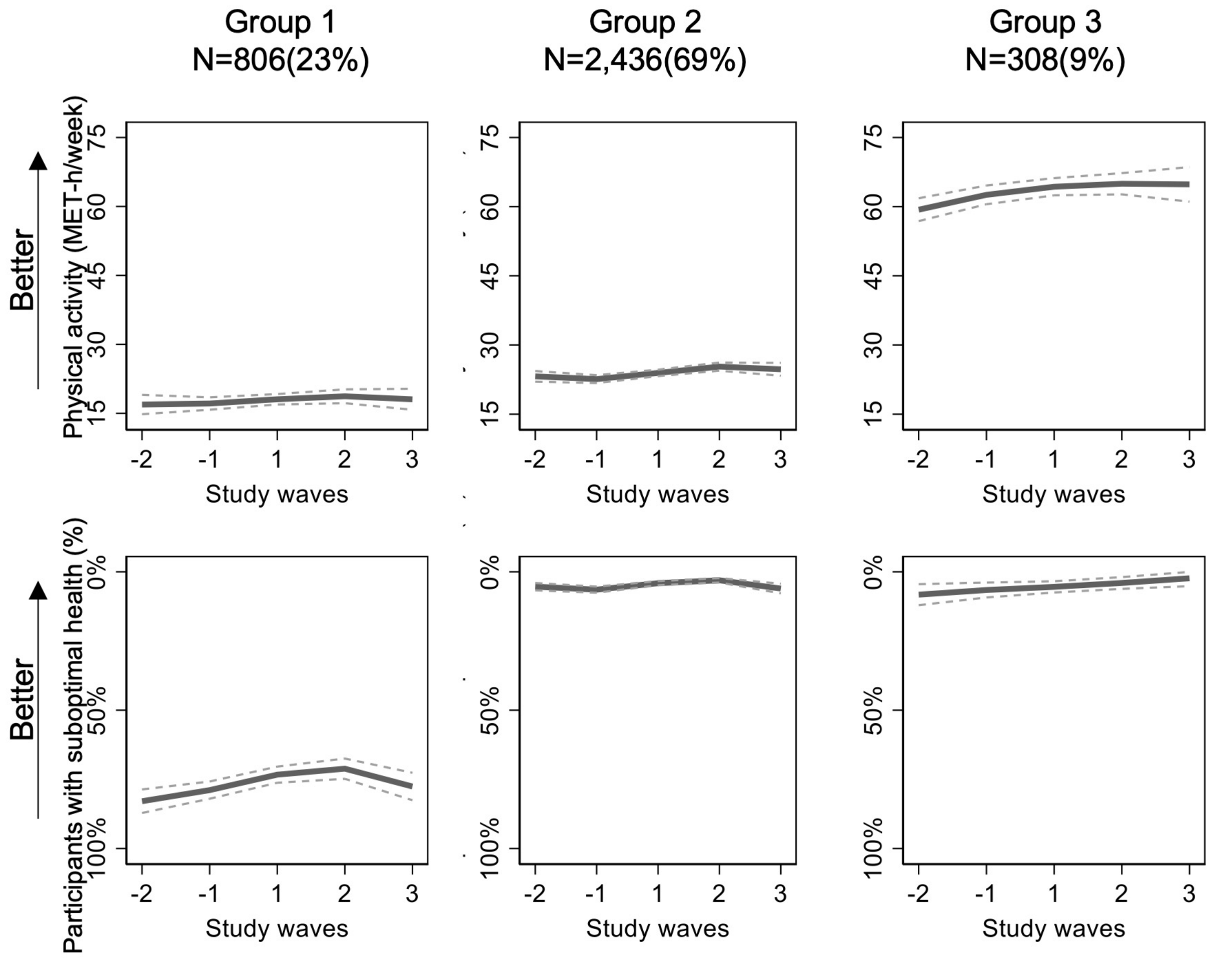

Supplement 2. The level of physical activity and physical functioning at different waves by trajectory groups

Study waves	Group #1:			Group #2:			Group #3:		
	Estimated mean	95% CI		Estimated mean	95% CI		Estimated mean	95% CI	
Physical activity, MET-h/week									
-2	16.9	14.8	19.0	23.2	22.1	24.4	59.4	56.9	61.8
-1	17.1	15.8	18.5	22.6	21.8	23.5	62.6	60.5	64.6
1	18.1	16.9	19.2	24.0	23.2	24.7	64.3	62.5	66.2
2	18.7	17.2	20.2	25.3	24.4	26.2	65.0	62.7	67.3
3	18.1	15.8	20.3	24.7	23.3	26.1	64.8	61.1	68.6
Percentage of participants with suboptimal self-rated health, %									
-2	83	79	87	5	4	7	8	4	12
-1	79	76	82	6	5	8	7	4	9
1	73	70	76	4	3	5	5	3	7
2	71	67	75	3	2	4	4	2	6
3	78	73	83	6	4	8	2	0	5

For peer review only

Supplement 3.

Trajectories of physical activity and self-rated health, self-rated health treated as a continuous variable.

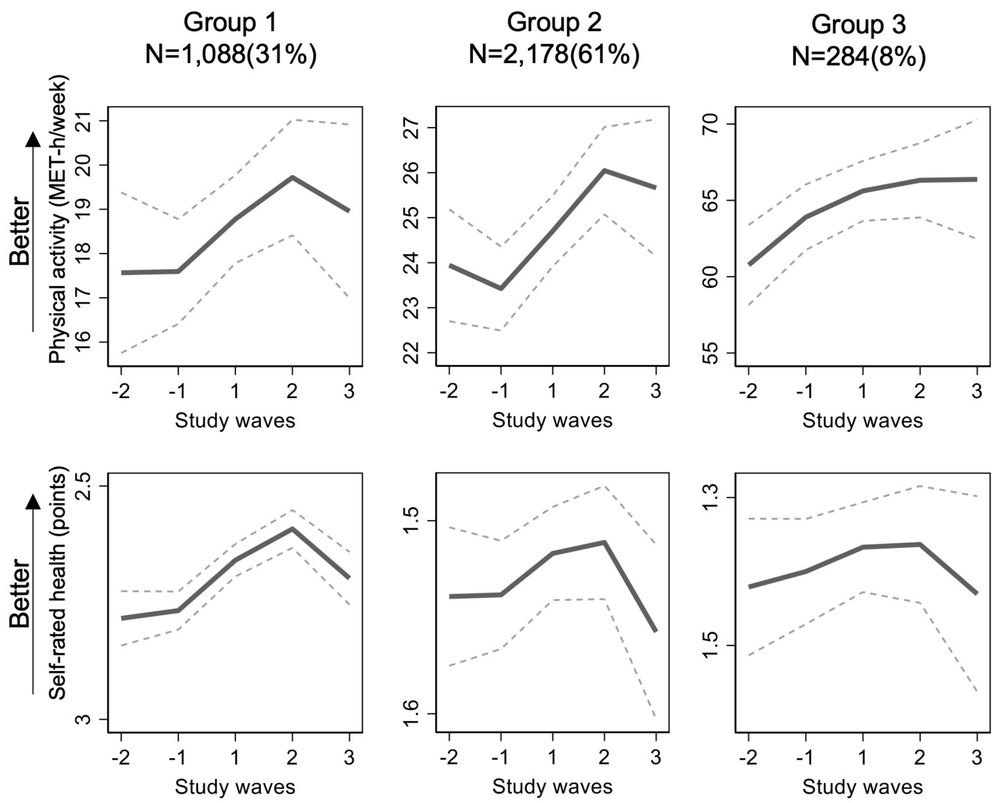

Goodness of fit of group-based trajectory analysis models, self-rated health treated as a continuous variable. The chosen model is shown in bold.

Number of clusters	Shape of trajectory	Smallest group		BIC[1]	AIC[2]	Smallest APP[3]
		N	%			
1	Cubic	3,550	100	75,810	76,769	1.0
2	Cubic	1,453	41	72,792	72,714	0.93
**3**	**Cubic**	**284**	**8**	**71,224**	**71,109**	**0.93**
4	Cubic	226	6	70,213	70,062	0.92

[1] BIC = Bayesian Information Criterion, [2] AIC = Akaike information criterion, [3] APP = Smallest average posterior probability

STROBE 2007 (v4) Statement—Checklist of items that should be included in reports of *cohort studies*

Section/Topic	Item #	Recommendation	Reported on page #
**Title and abstract**	1	(*a*) Indicate the study's design with a commonly used term in the title or the abstract	1
		(*b*) Provide in the abstract an informative and balanced summary of what was done and what was found	3
**Introduction**			
Background/rationale	2	Explain the scientific background and rationale for the investigation being reported	6,7
Objectives	3	State specific objectives, including any prespecified hypotheses	7
**Methods**			
Study design	4	Present key elements of study design early in the paper	8
Setting	5	Describe the setting, locations, and relevant dates, including periods of recruitment, exposure, follow-up, and data collection	
Participants	6	(*a*) Give the eligibility criteria, and the sources and methods of selection of participants. Describe methods of follow-up	8
		(*b*) For matched studies, give matching criteria and number of exposed and unexposed	
Variables	7	Clearly define all outcomes, exposures, predictors, potential confounders, and effect modifiers. Give diagnostic criteria, if applicable	8–10
Data sources/ measurement	8*	For each variable of interest, give sources of data and details of methods of assessment (measurement). Describe comparability of assessment methods if there is more than one group	8–10
Bias	9	Describe any efforts to address potential sources of bias	10,11, 17
Study size	10	Explain how the study size was arrived at	8
Quantitative variables	11	Explain how quantitative variables were handled in the analyses. If applicable, describe which groupings were chosen and why	8,9
Statistical methods	12	(*a*) Describe all statistical methods, including those used to control for confounding	10,11
		(*b*) Describe any methods used to examine subgroups and interactions	10,11
		(*c*) Explain how missing data were addressed	
		(*d*) If applicable, explain how loss to follow-up was addressed	
		(*e*) Describe any sensitivity analyses	11
**Results**			

For peer review only

Participants	13*	(a) Report numbers of individuals at each stage of study—eg numbers potentially eligible, examined for eligibility, confirmed eligible, included in the study, completing follow-up, and analysed	8
		(b) Give reasons for non-participation at each stage	
		(c) Consider use of a flow diagram	
Descriptive data	14*	(a) Give characteristics of study participants (eg demographic, clinical, social) and information on exposures and potential confounders	12, table 1
		(b) Indicate number of participants with missing data for each variable of interest	
		(c) Summarise follow-up time (eg, average and total amount)	
Outcome data	15*	Report numbers of outcome events or summary measures over time	12-14
Main results	16	(*a*) Give unadjusted estimates and, if applicable, confounder-adjusted estimates and their precision (eg, 95% confidence interval). Make clear which confounders were adjusted for and why they were included	12-14
		(*b*) Report category boundaries when continuous variables were categorized	8, 9
		(*c*) If relevant, consider translating estimates of relative risk into absolute risk for a meaningful time period	
Other analyses	17	Report other analyses done—eg analyses of subgroups and interactions, and sensitivity analyses	12-14
**Discussion**			
Key results	18	Summarise key results with reference to study objectives	15
**Limitations**			
Interpretation	20	Give a cautious overall interpretation of results considering objectives, limitations, multiplicity of analyses, results from similar studies, and other relevant evidence	15-17
Generalisability	21	Discuss the generalisability (external validity) of the study results	17
**Other information**			
Funding	22	Give the source of funding and the role of the funders for the present study and, if applicable, for the original study on which the present article is based	2

*Give information separately for cases and controls in case-control studies and, if applicable, for exposed and unexposed groups in cohort and cross-sectional studies.

Note: An Explanation and Elaboration article discusses each checklist item and gives methodological background and published examples of transparent reporting. The STROBE checklist is best used in conjunction with this article (freely available on the Web sites of PLoS Medicine at http://www.plosmedicine.org/, Annals of Internal Medicine at http://www.annals.org/, and Epidemiology at http://www.epidem.com/). Information on the STROBE Initiative is available at www.strobe-statement.org.

For peer review only

BMJ Open

Physical activity and self-rated health during retirement transition – a multi-trajectory analysis of concurrent changes among public sector employees

Journal:	*BMJ Open*
Manuscript ID	bmjopen-2023-073876.R1
Article Type:	Original research
Date Submitted by the Author:	09-Aug-2023

Complete List of Authors:	Lintuaho, Roosa; TYKS Turku University Hospital, Department of Emergency Medicine; TYKS Turku University Hospital, Department of Physical and Rehabilitation Medicine<br>Saltychev, Mikhail; TYKS Turku University Hospital, Department of Physical and Rehabilitation Medicine<br>Pentti, Jaana; University of Turku, Department of Public Health; University of Helsinki Clinicum Unit<br>Vahtera, Jussi; University of Turku, Department of Public Health; University of Turku, Centre for Population Health Research<br>Stenholm, Sari; University of Turku, Department of Public Health; University of Turku, Centre for Population Health Research

**Primary Subject Heading**:	Public health
Secondary Subject Heading:	Sports and exercise medicine
Keywords:	PUBLIC HEALTH, Aging, Life Change Events

SCHOLARONE™
Manuscripts

I, the Submitting Author has the right to grant and does grant on behalf of all authors of the Work (as defined in the below author licence), an exclusive licence and/or a non-exclusive licence for contributions from authors who are: i) UK Crown employees; ii) where BMJ has agreed a CC-BY licence shall apply, and/or iii) in accordance with the terms applicable for US Federal Government officers or employees acting as part of their official duties; on a worldwide, perpetual, irrevocable, royalty-free basis to BMJ Publishing Group Ltd ("BMJ") its licensees and where the relevant Journal is co-owned by BMJ to the co-owners of the Journal, to publish the Work in this journal and any other BMJ products and to exploit all rights, as set out in our licence.

The Submitting Author accepts and understands that any supply made under these terms is made by BMJ to the Submitting Author unless you are acting as an employee on behalf of your employer or a postgraduate student of an affiliated institution which is paying any applicable article publishing charge ("APC") for Open Access articles. Where the Submitting Author wishes to make the Work available on an Open Access basis (and intends to pay the relevant APC), the terms of reuse of such Open Access shall be governed by a Creative Commons licence – details of these licences and which Creative Commons licence will apply to this Work are set out in our licence referred to above.

Other than as permitted in any relevant BMJ Author's Self Archiving Policies, I confirm this Work has not been accepted for publication elsewhere, is not being considered for publication elsewhere and does not duplicate material already published. I confirm all authors consent to publication of this Work and authorise the granting of this licence.

For peer review only

Physical activity and self-rated health during retirement transition – a multi-trajectory analysis of concurrent changes among public sector employees

Roosa Lintuaho[1,2], Mikhail Saltychev[2], Jaana Pentti[3,4,5], Jussi Vahtera[3,4], Sari Stenholm[3,4, 6]

[1] Department of Emergency Medicine, Turku University Hospital and University of Turku, Finland

[2] Department of Physical and Rehabilitation Medicine, Turku University Hospital and University of Turku, Turku, Finland

[3] Department of Public Health, University of Turku and Turku University Hospital, Turku, Finland

[4] Centre for Population Health Research, University of Turku and Turku University Hospital; Turku, Finland

[5] Clinicum, Faculty of Medicine, University of Helsinki, Helsinki, Finland

[6] Research Services, Turku University Hospital and University of Turku, Finland

Corresponding author: Roosa Lintuaho, roemti@utu.fi

Word count: 3025

For peer review only

For peer review only - http://bmjopen.bmj.com/site/about/guidelines.xhtml

ABSTRACT

Objectives: The aim of the study was to evaluate concurrent changes in physical activity and self-rated health during retirement transition over four years by multivariate trajectory analysis and to examine, whether sociodemographic and lifestyle factors predict the probability of being classified to a certain subgroup of observed changes.

Design: prospective cohort study

Setting: public sector employees

Participants: 3,550 participants of the Finnish Retirement and Aging (FIREA) study

Primary and secondary outcome measures: Participants estimated on a yearly questionnaire their weekly hours of different types of activities converted to MET-h/week. Self-rated health was assessed on a 5-point Likert-like scale from poor to excellent and dichotomized as suboptimal and optimal. Multivariate trajectory analysis was used to distinguish different subgroups of trajectories. Multinomial regression analysis was used to describe the associations between covariates and the probability of being classified to a certain trajectory group.

Results: Three trajectory groups were identified, all displaying increasing activity during retirement with a simultaneous decrease in perceived suboptimal health. Physical activity peaked at 18 months after retirement and then slightly decreased, except for initially highly physically active participants (9%) with good self-rated health, who demonstrated constant high level of physical activity. Male gender, professional occupation, being married or co-habiting, BMI <30kg/m^2, not smoking and using alcohol below risk levels were associated with higher physical activity and better self-rated health.

Conclusion: Changes in physical activity and perceived health during retirement transition were interconnected. Both were improved during retirement transition, but the change was temporary. Longer follow-up studies are required to assess the changes over longer period after retirement.

For peer review only

Keywords: retirement; physical activity; self-rated health

For peer review only

For peer review only - http://bmjopen.bmj.com/site/about/guidelines.xhtml

Strengths and limitations of this study

- A data-driven approach to finding subgroups with different trajectory paths.
- Large sample size, representative of the public sector workforce.
- Repeated measurements of self-rated health and physical activity.
- The self-reported nature of the variables might lead to information bias.
- Participants were mostly retiring due to old age, leaving out those retiring due to health reasons, who might get different results.

For peer review only

For peer review only - http://bmjopen.bmj.com/site/about/guidelines.xhtml

INTRODUCTION

Retirement may change many daily routines, such as e.g., diet, sleeping rhythm, psychological well-being, as well as the types and intensity of leisure-time activities or hobbies.[1-4] Such a comprehensive change may also affect health perception.

Several studies have reported increasing physical activity during the retirement transition.[1, 5-12] According to multiple follow-up studies, this increase might be temporary.[6, 10, 13] The type of retirement affects the change in physical activity – retiring due to health reasons has been associated with lesser increase or even decreasing physical activity after retirement.[7, 9] Gender, work characteristics and socioeconomic status may also moderate these changes. Studies by Touvier et al.[14] and Lahti et al.[7] have reported greater increase in physical activity during retirement transition among men, whereas study by Sjösten et al.[5] has showed greater increase in physical activity among retiring women. Retiring from non-manual work seems to be beneficial regarding physical activity comparing with retiring from manual jobs.[6, 15, 16] Systematic reviews have also reported the effect of socioeconomic status on the intensity of physical activity after retirement – higher status has been associated with improved physical activity.[1, 17] Some sex-related differences have been reported concerning the effect of occupational status – women retiring from manual work have showed decreased accelerometer-measured physical activity, while physical activity has not changed among women retiring from non-manual work.[16] Respectively, men retiring from non-manual work had increased physical activity, while those retiring from manual work have not demonstrated any change.[16]

Previous knowledge on the change in self-rated health during retirement transition has been inconsistent. In a French study among employees, who retired at relatively young age (54 years on average), perceived health has improved after retirement, especially for those with poorer working conditions and health problems prior to retirement.[18] In a systematic review, mental health has

For peer review only - http://bmjopen.bmj.com/site/about/guidelines.xhtml

often substantially improved after retirement. Instead, the change in self-rated health has been less clear as different studies have reported improvement, deterioration or no change in self-rated health.[19] A meta-analysis by van der Heide et al. has reported either deterioration or improvement in self-rated health depending on which set of original studies has been pooled.[19] It seems that retirement due to health reasons is associated with greater positive changes in perceived health compared with those who retired due to old age.[19] Worsening physical health has been associated with lower occupational status, manual work and higher prevalence of chronic conditions.[20] In another study, job strain and physically demanding work have been associated with suboptimal health prior to retirement. In turn, sustained good health has been associated with normal weight, non-smoking and higher physical activity.[21]

Previous research has found associations between physical activity and self-rated health.[21, 22] Thus, it can be assumed that changes in physical activity and self-rated health may follow the same patterns around the retirement transition. Simultaneous changes on the subject have not been studied before.

The aim of this study was to evaluate concurrent changes in physical activity and self-rated health during retirement transition among Finnish public sector employees by using a multi-trajectory analysis to identify subgroups with different trajectory paths during this transition.

For peer review only

For peer review only - http://bmjopen.bmj.com/site/about/guidelines.xhtml

METHODS

Study population

The Finnish Retirement and Aging Study (FIREA) is an ongoing longitudinal cohort study, which follows aging workers from final years in working life to full-time retirement and until old age.[23] The eligible population for the FIREA included all public sector employees whose individual retirement date was between 2014 and 2019 and who were working in the year 2012 in one of 27 municipalities, nine cities or five hospital districts included in the study. Municipalities and cities, involved in the study, are responsible for school education, daycare centers, social services, libraries, maintaining roads, and primary healthcare services, among others. Respectively, hospital districts, involved in the study, are responsible for providing secondary healthcare services. Thus, the eligible population covered a wide variety of occupations.

The participants were first contacted 18 months prior to their estimated retirement date, which was obtained from the register kept by the pension provider for public sector employees (Keva). The actual retirement date was self-reported. The participants received a questionnaire, which has been thereafter sent annually, at least four times, expanding the follow-up 2.5 years after retirement. Participants who had provided information regarding physical activity and self-rated health at least two consecutive times, one right before retirement and one after, were included in the current study (n=3,550). There were two possible survey waves before the retirement, waves -2 and -1, and three possible waves after the retirement, waves 1, 2 and 3 (Figure 1).

All the respondents have provided a written informed consent. The FIREA is following the Declaration of Helsinki and has been approved by the Ethics Committee of Hospital District of Southwest Finland.

For peer review only - http://bmjopen.bmj.com/site/about/guidelines.xhtml

Measurement of physical activity and self-rated health

Physical activity was self-reported at each study wave. The participants were asked to estimate their average weekly hours of leisure-time physical activity (including active travel to work) in activities comparable to walking, brisk walking, jogging or running.[24] The level of physical activity was then converted into metabolic equivalent of task (MET), which describes the amount of consumed energy comparing to resting. One MET unit of 3.5 ml/kg/minute corresponds to oxygen consumption while sitting at rest. Weekly physical activity was expressed as MET-h/week. To help the interpretation of the results, following categorization was used: low (<14 MET-h/week), moderate (14 to <30 MET-h/week) or high (≥30 MET-h/week) physical activity.[24, 25] This categorization was chosen since physical activity lower than 14 MET-h/week (approximately the equivalent of 140 minutes of brisk walking) has been reported to be associated with higher risk of cardiovascular disease[26] and the activity level of 30 MET-h/week (the equivalent of 300 minutes of brisk walking) has been shown to be needed for weight management.[27]

Self-reported health was inquired at each study wave. Participants were asked to rate their health on a five-point Likert-like scale:1-good, 2-rather good, 3-average, 4-rather poor or 5-poor. For the purpose of analysis, the result was dichotomized as 3-5 as "suboptimal health" vs. 1-2 as "optimal health".

For peer review only

Independent variables

Age was defined in years. Marital status was dichotomized as "married or co-habiting" vs. "single". Occupational titles were obtained from the register of pension provider (Keva), and they were coded according to the International Standard Classification of Occupations (ISCO) and dichotomized as "professionals" (ISCO major groups 1 – 4) vs. "service and manual workers" (ISCO major groups 5 –

For peer review only - http://bmjopen.bmj.com/site/about/guidelines.xhtml

9). Body Mass Index (BMI) was calculated as weight/height^2 according to self-reported body height and weight. BMI was dichotomized as “normal and overweight” (BMI < 30kg/m^2) vs. “obesity” (≥30kg/m^2). Smoking was dichotomized as “current smokers” vs. “never-smokers and former smokers”. Alcohol consumption was obtained from the survey as units consumed weekly and converted into grams of pure alcohol per week (g/week). Amount of >288 g/week for men and >192 g/week for women were considered a cut-off for excess alcohol consumption and dichotomized as “risk-users “vs. “non-risk-users”. Information on covariates were obtained at the last available wave before retirement, wave -1.

Statistical analysis

The covariates were reported as means and standard deviations or as absolute numbers and percentage, when appropriate.

Group-based multi-trajectory analysis (GBTA) was used to investigate the developmental trajectories (a course of outcome over time) of physical activity and self-reported health. The group-based multi-trajectory analysis is a form of finite mixture modelling for analysing longitudinal repeated measures data.[28] It is a data-driven form of analysis which is able to distinguish and describe subpopulations (clusters) existing within a studied population, while conventional statistics only show the average outcome over time. The trajectories of such subpopulations may differ substantially from each other and from the average trajectory observed in the entire population. The censored (known also as ‘regular’) normal model of group-based multi-trajectory analysis was used. The goodness of model fit was judged by running the procedure several times with a number of clusters starting from one up to four. The Bayesian Information Criterion (BIC), Akaike information criterion (AIC) and average posterior probability (APP) were used

For peer review only - http://bmjopen.bmj.com/site/about/guidelines.xhtml

as criteria to confirm the goodness of fit. Linear, quadratic and cubic regression models were tested and cubic model was retained for using in the analysis. The cut-off for the smallest group was set at ≥5% of the entire cohort. Physical activity was treated as a continuous variable. Self-rated health was dichotomized in the analysis as suboptimal (yes/no). For sensitivity analysis, self-rated health was also used as a continuous variable.

Multinomial regression analysis was used to describe the associations of demographic and lifestyle factors and probability of being classified into a particular cluster. The results were presented as risk ratios (RRs) and their 95% confidence intervals (95% Cis). The RRs were adjusted for age and gender, except for the RR for gender which was only adjusted for age.

The analyses were performed using Stata/IC Statistical Software: Release 17. College Station (StataCorp LP, TX, USA). The additional freely available Stata module 'traj' was required to conduct group-based trajectory analysis.[29]

Patient and public involvement

None.

For peer review only

For peer review only - http://bmjopen.bmj.com/site/about/guidelines.xhtml

RESULTS

The characteristics of the study sample prior to retirement (study wave -1) are presented in Table 1. Of the respondents, 83% were women, 31% were single and 65% were professional workers. Mean age before the retirement was 63.4 (SD 1.4) years. Only 9% were currently smoking, 2% consumed alcohol over risk limits and 28% had a BMI >=30kg/m^2. Of the respondents, 24% rated their health as suboptimal and 22% reported low, 18% moderate and 60% high physical activity.

For peer review only

Trajectory groups

Three trajectories of concurrent changes in physical activity and self-rated health were identified (Figure 2). The three-cluster model was chosen, since the smallest group in the next, four-cluster model, fell below the pre-defined cut-off of 5% (Table 2). The smallest APP was sufficient 0.93 for the three-cluster model. Compared to the two-cluster model, a three-cluster model demonstrated BIC and AIC values closer to zero. The results are also presented using the same scale for a Y-axis in Supplement 1. The estimates of physical activity and self-rated health by trajectory group at each wave are presented in Supplement 2.

Group #1: Moderate physical activity and suboptimal self-rated health (23%)

Physical activity was at a moderate level of 17.1 MET-h/week six months before retirement. First, it slightly increased from final working years until 18 months after retirement up to 18.1 MET-h/week and thereafter slightly decreased. The trajectory of self-rated health was mirroring the changes in physical activity: 79% reported suboptimal health six months prior to retirement improving down to 71% 18 months after the retirement and then slightly worsening again.

Group #2: Moderate physical activity and good self-rated health (69%)

Physical activity was at a moderate level of 22.6 MET-h/week six months before retirement and increased during retirement transition all the way to 18 months after the retirement to 24.4. MET-

For peer review only - http://bmjopen.bmj.com/site/about/guidelines.xhtml

h/week. After that, physical activity showed a mild decrease staying, however, higher than the initial level. In this group, only 6% of the respondent reported suboptimal health six months before the retirement and decreasing down to 3% on the way to 18 months after the retirement. After that time point, the percentage of people with suboptimal health grew again up to 6%.

Group #3: High physical activity and good self-rated health (9%)

This smallest group demonstrated high level of physical activity and good self-rated health during the entire follow-up. The intensity of physical activity increased from 62.6 MET-h/week six months before retirement up to 65 MET-h/week 18 months after retirement. There was no decline in physical activity, unlike in the other two groups. Respectively, the portion of respondents with suboptimal health decreased from 7% down to 4%.

Sensitivity analysis

The trajectory solution was similar when the analysis was rerun treating self-rated health as a continuous variable instead of binary variable (Supplement 3). The respective groups were:

Group #1: moderate physical activity and suboptimal self-rated health (31%)

Group #2: moderate physical activity and good self-rated health (61%)

Group #3: increasing high physical activity and good self-rated health (8%).

Associations between sociodemographic and health behavioural factors and trajectory groups

Compared to group #1 (moderate physical activity and suboptimal self-rated health), in groups #2(moderate physical activity and good self-rated health) and #3 (increasing physical activity and improving self-rated health), there were more married or co-habiting persons (RR 1.23, 95%CI 1.04-1.47/RR 1.48, 95% CI 1.10-1.99), fewer manual workers (RR 0.77, 95%CI 0.65-0.91/RR 0.66, 95% CI 0.50-0.88), fewer overweight or obese participants (RR 0.35, 95%CI 0.30-042/RR 0.13, 95% CI 0.09-

For peer review only - http://bmjopen.bmj.com/site/about/guidelines.xhtml

0.19), fewer smokers (RR 0.68, 95%CI 0.52-0.88/RR 0.31, 95% CI 0.17-0.58) and fewer risk-users of alcohol (RR 0.60, 95% CI 0.38-0.96/RR 0.23, 95% CI 0.07-0.77). (Table 3)

When comparing the most active and healthy groups #2 and #3, there were fewer women (RR 0.63, 95% CI 0.47-0.84), fewer persons with BMI >30 kg/m^2 (RR 0.36, 95% CI 0.25-0.53) and smokers (RR 0.46, 95% CI 0.25-0.83) in group #3 than in group #2 (Table 3).

For peer review only

For peer review only - http://bmjopen.bmj.com/site/about/guidelines.xhtml

Discussion

In this prospective cohort study among 3,550 public sector employees, three trajectory groups of concurrent changes in physical activity and self-rated health were identified. Overall, in all three groups, physical activity and self-rated health were clearly interconnected; trajectories for both parameters were mirroring each other – increase in physical activity was accompanied by improving health perception and vice versa. Higher physical activity was clearly associated with better self-rated health. These findings mirror previous reports on association between higher physical activity and better self-rated health.[22, 30, 31] However, concurrent trajectories of changes in these two parameters have not been studied before.

Overall, physical activity and self-rated health increased simultaneously during retirement transition. For 91% of the participants, this increase was only temporary. In regards of physical activity, this has been seen in previous research as well[6, 10, 13]. Only in a small proportion of participants, who were initially highly active and in good health, the improvement was maintained after the retirement transition throughout the entire follow-up. In this group, there were more men, professional workers and participants with BMI <30 kg/m^2 than in other groups, which is in line with previous findings; higher physical activity after retirement has been associated with higher socioeconomical status[1, 12, 17], normal weight[12] and in some studies, male sex[7, 14].

Some previous studies have observed different paths of self-rated health. A study from New Zealand, identified three trajectories of self-rated health: declining, recovering and one with rather stable self-rated health that has showed only a minor decrease after retirement.[20] In a Finnish study, four trajectories around retirement transition were observed: two with sustained level of health, either good or suboptimal, one with improving self-rated health and one with declining health.[21] The difference in these results compared to current could be due to the difference in

For peer review only

study samples, but also due to the multi-trajectory-setting of the current study, in which changes in physical activity are just as important when defining the trajectories.

Compared to the group with moderate activity and poor self-rated health, there were more professional workers, persons with normal weight, non-smokers, co-habiting participants and participants who consume alcohol within moderation in the other two groups with higher activity and better self-rated health. These findings are in line with previous work; higher physical activity after retirement has been associated with retiring form professional work and higher socioeconomical status[1, 6, 17] and lower BMI[12]. Poorer self-rated health has also been found to be associated with lower socioeconomic status, overweight and lower physical activity,[21, 32] and good self-rated health has been associated with normal weight, non-smoking and normal level of physical activity.[21]

Although there was improvement in all groups regarding physical activity and self-rated health, the improvement in self-rated health was most prominent in the group with lowest activity and poorer self-rated health; the proportion of participants with suboptimal health decreased by 12%, whereas in other groups the change was 3 to 6% during follow-up (although due to small percentage of individuals experiencing suboptimal health in these groups, such an improvement wasn't possible). In this group, there was more single people, manual workers, participants with obesity, current smokers, and participants with risk-use of alcohol. This has been seen in other studies as well; in the New Zealand study, retirement has been most beneficial in regards of self-rated health to those with manual occupations, low to medium socioeconomic statuses and multiple chronic conditions.[20] On the other hand, some studies have found retirement to be associated with declining self-rated health for people with higher socioeconomic status, which was not the case in the current study.[19, 20]

For peer review only - http://bmjopen.bmj.com/site/about/guidelines.xhtml

Strengths and limitations

This study was conducted on a large cohort, representative of the public sector workforce. Strengths of this study also include a data-driven approach to defining clusters and repeated measurements of physical activity and self-rated health.

There are some limitations as well, which should be considered. The participants were, due to the inclusion criteria, mostly retiring due to old age. Since most of the participants could keep on working until their retirement age, they were probably healthier and in better shape than those who retired earlier due to health reasons. Although the sample is representative of public sector employees in Finland, the sample was predominated by women (83%) and professional workers (65%), which affects the generalisability of these findings into general population. This study only assessed leisure-time and active travel to work, leaving out work-related physical activity. The self-reported nature of the estimates could lead to information bias. Self-rated health is, although, a widely used measurement perceived as a valid indicator of sustaining well-being. It is an independent predictor of mortality[33], as well as increased use of prescribed medications and healthcare and social services[34]. To minimize the risk of bias due to the measurement used, the analyses were conducted separately with self-rated health treated as a dichotomized variable and as a continuous variable.

Further research is needed on interventions on how to maintain the positive trend in self-rated health and physical activity in the years following retirement.

Conclusions

Physical activity was found to increase, and self-rated health improve during retirement transition, although this change was temporary for most participants. Changes in physical activity and self-rated health seemed to be interconnected. Lower physical activity, being single, manual work,

For peer review only

obesity, current smoking, and risk-use of alcohol were associated with poorer self-rated health and lower physical activity.

For peer review only

For peer review only - http://bmjopen.bmj.com/site/about/guidelines.xhtml

Captions for figures

Figure 1. Study waves and time in relation to retirement

Figure 2. Trajectories of physical activity and self-rated health during retirement transition. The scale is different in each group.

For peer review only

For peer review only - http://bmjopen.bmj.com/site/about/guidelines.xhtml

DECLARATIONS

Ethics approval and consent to participate: A written informed consent was obtained from all the participants. The study was approved by the Ethics Committee of Hospital District of Southwest Finland.

Consent for publication: Not applicable

Availability of data and material: Anonymised partial datasets of the Finnish Retirement and Aging Study are available by application with bona fide researchers with an established scientific record and bona fide organisations. For more information, please contact Professor Sari Stenholm (firea@utu.fi).

Competing interests: None

Funding: This study was supported by funding granted by the Academy of Finland (321409 and 329240 to JV, 286294, 319246, 294154, 332030 to SS), Finnish Ministry of Education and Culture (to SS); Juho Vainio Foundation (to SS), and Competitive State Research Financing of the Expert Responsibility Area of the Turku University Hospital (to RL and SS)

Author statement: All the authors (RL, MS, JP, JV, SS) substantially contributed to the conception and design of the work, the interpretation of the results and revising it critically for important intellectual content. SS and JV were responsible for the acquisition of data for the work. MS and JP were responsible for the statistical analysis. RL was responsible for drafting the work. All the authors (RL, MS, JP, JV, SS) have finally approved the version to be published and they are agreed to be accountable for all aspects of the work in ensuring that questions related to the accuracy or integrity of any part of the work are appropriately investigated and resolved.

Acknowledgements: None

For peer review only

For peer review only

For peer review only - http://bmjopen.bmj.com/site/about/guidelines.xhtml

References

1. Barnett I, van Sluijs EM, Ogilvie D. Physical activity and transitioning to retirement: a systematic review. Am J Prev Med. 2012;43(3):329-36. Epub 2012/08/18. doi: 10.1016/j.amepre.2012.05.026. PubMed PMID: 22898127; PubMed Central PMCID: PMCPMC3830178.

2. Ali-Kovero K, Pietilainen O, Mauramo E, Jappinen S, Rahkonen O, Lallukka T, et al. Changes in fruit, vegetable and fish consumption after statutory retirement: a prospective cohort study. Br J Nutr. 2020;123(12):1390-5. Epub 20200120. doi: 10.1017/S0007114520000136. PubMed PMID: 31955724.

3. Vahtera J, Westerlund H, Hall M, Sjosten N, Kivimaki M, Sal OP, et al. Effect of retirement on sleep disturbances: the GAZEL prospective cohort study. Sleep. 2009;32(11):1459-66. doi: 10.1093/sleep/32.11.1459. PubMed PMID: 19928385; PubMed Central PMCID: PMCPMC2768952.

4. Lahdenpera M, Virtanen M, Myllyntausta S, Pentti J, Vahtera J, Stenholm S. Psychological Distress During the Retirement Transition and the Role of Psychosocial Working Conditions and Social Living Environment. J Gerontol B Psychol Sci Soc Sci. 2022;77(1):135-48. doi: 10.1093/geronb/gbab054. PubMed PMID: 34396418; PubMed Central PMCID: PMCPMC8755891.

5. Sjosten N, Kivimaki M, Singh-Manoux A, Ferrie JE, Goldberg M, Zins M, et al. Change in physical activity and weight in relation to retirement: the French GAZEL Cohort Study. BMJ Open. 2012;2:e000522. Epub 2012/02/10. doi: 10.1136/bmjopen-2011-000522. PubMed PMID: 22318663; PubMed Central PMCID: PMCPMC3277904.

6. Stenholm S, Pulakka A, Kawachi I, Oksanen T, Halonen JI, Aalto V, et al. Changes in physical activity during transition to retirement: a cohort study. Int J Behav Nutr Phys Act. 2016;13:51. Epub 2016/04/17. doi: 10.1186/s12966-016-0375-9. PubMed PMID: 27084334; PubMed Central PMCID: PMCPMC4833915.

7. Lahti J, Holstila A, Manty M, Lahelma E, Rahkonen O. Changes in leisure time physical activity and subsequent disability retirement: A register-linked cohort study. Int J Behav Nutr Phys Act. 2016;13(1):99. Epub 20160906. doi: 10.1186/s12966-016-0426-2. PubMed PMID: 27599466; PubMed Central PMCID: PMCPMC5012048.

8. Engberg E, Alen M, Kukkonen-Harjula K, Peltonen JE, Tikkanen HO, Pekkarinen H. Life events and change in leisure time physical activity: a systematic review. Sports Med. 2012;42(5):433-47. doi: 10.2165/11597610-000000000-00000. PubMed PMID: 22512413.

9. Feng X, Croteau K, Kolt GS, Astell-Burt T. Does retirement mean more physical activity? A longitudinal study. BMC Public Health. 2016;16:605. Epub 20160720. doi: 10.1186/s12889-016-3253-0. PubMed PMID: 27439914; PubMed Central PMCID: PMCPMC4955250.

10. Holstila A, Manty M, Rahkonen O, Lahelma E, Lahti J. Statutory retirement and changes in self-reported leisure-time physical activity: a follow-up study with three time-points. BMC Public Health. 2017;17(1):528. Epub 20170530. doi: 10.1186/s12889-017-4455-9. PubMed PMID: 28558730; PubMed Central PMCID: PMCPMC5450199.

11. Henning G, Stenling A, Bielak AAM, Bjalkebring P, Gow AJ, Kivi M, et al. Towards an active and happy retirement? Changes in leisure activity and depressive symptoms during the retirement transition. Aging Ment Health. 2021;25(4):621-31. Epub 20200122. doi: 10.1080/13607863.2019.1709156. PubMed PMID: 31965817.

12. Lintuaho R, Saltychev M, Pentti J, Vahtera J, Stenholm S. Multi-trajectory analysis of changes in physical activity and body mass index in relation to retirement: Finnish Retirement and

For peer review only - http://bmjopen.bmj.com/site/about/guidelines.xhtml

Aging study. PLoS One. 2022;17(12):e0278405. Epub 20221201. doi: 10.1371/journal.pone.0278405. PubMed PMID: 36454722; PubMed Central PMCID: PMCPMC9714751.
13. Van Dyck D, Cardon G, De Bourdeaudhuij I. Longitudinal changes in physical activity and sedentary time in adults around retirement age: what is the moderating role of retirement status, gender and educational level? BMC Public Health. 2016;16(1):1125. Epub 20161028. doi: 10.1186/s12889-016-3792-4. PubMed PMID: 27793134; PubMed Central PMCID: PMCPMC5084354.
14. Touvier M, Bertrais S, Charreire H, Vergnaud AC, Hercberg S, Oppert JM. Changes in leisure-time physical activity and sedentary behaviour at retirement: a prospective study in middle-aged French subjects. Int J Behav Nutr Phys Act. 2010;7:14. Epub 20100204. doi: 10.1186/1479-5868-7-14. PubMed PMID: 20181088; PubMed Central PMCID: PMCPMC2834610.
15. Chung S, Domino ME, Stearns SC, Popkin BM. Retirement and physical activity: analyses by occupation and wealth. Am J Prev Med. 2009;36(5):422-8. Epub 20090306. doi: 10.1016/j.amepre.2009.01.026. PubMed PMID: 19269129.
16. Pulakka A, Leskinen T, Suorsa K, Pentti J, Halonen JI, Vahtera J, et al. Physical Activity across Retirement Transition by Occupation and Mode of Commute. Med Sci Sports Exerc. 2020;52(9):1900-7. Epub 2020/03/10. doi: 10.1249/MSS.0000000000002326. PubMed PMID: 32150014; PubMed Central PMCID: PMCPMC7431137.
17. Vansweevelt N, Boen F, van Uffelen J, Seghers J. Socioeconomic Differences in Physical Activity and Sedentary Behavior During the Retirement Transition: A Systematic Review of Longitudinal Studies. J Phys Act Health. 2022:1-15. Epub 20220819. doi: 10.1123/jpah.2022-0196. PubMed PMID: 35985645.
18. Westerlund H, Kivimaki M, Singh-Manoux A, Melchior M, Ferrie JE, Pentti J, et al. Self-rated health before and after retirement in France (GAZEL): a cohort study. Lancet. 2009;374(9705):1889-96. Epub 20091110. doi: 10.1016/S0140-6736(09)61570-1. PubMed PMID: 19897238.
19. van der Heide I, van Rijn RM, Robroek SJ, Burdorf A, Proper KI. Is retirement good for your health? A systematic review of longitudinal studies. BMC Public Health. 2013;13:1180. Epub 20131213. doi: 10.1186/1471-2458-13-1180. PubMed PMID: 24330730; PubMed Central PMCID: PMCPMC4029767.
20. Szabo A, Allen J, Stephens C, Alpass F. Is retirement associated with physical health benefits? A longitudinal investigation with older New Zealanders. Age Ageing. 2019;48(2):267-72. doi: 10.1093/ageing/afy176. PubMed PMID: 30379990.
21. Stenholm S, Virtanen M, Pentti J, Oksanen T, Kivimaki M, Vahtera J. Trajectories of self-rated health before and after retirement: evidence from two cohort studies. Occup Environ Med. 2020;77(2):70-6. Epub 20191211. doi: 10.1136/oemed-2019-106026. PubMed PMID: 31826927.
22. Sperlich S, Beller J, Epping J, Tetzlaff J, Geyer S. Trends in self-rated health among the elderly population in Germany from 1995 to 2015 - the influence of temporal change in leisure time physical activity. BMC Public Health. 2020;20(1):113. Epub 20200128. doi: 10.1186/s12889-020-8218-7. PubMed PMID: 31992257; PubMed Central PMCID: PMCPMC6988275.
23. Leskinen T, Pulakka A, Heinonen OJ, Pentti J, Kivimaki M, Vahtera J, et al. Changes in non-occupational sedentary behaviours across the retirement transition: the Finnish Retirement and Aging (FIREA) study. J Epidemiol Community Health. 2018;72(8):695-701. Epub 20180410. doi: 10.1136/jech-2017-209958. PubMed PMID: 29636399.

For peer review only

24. Kujala UM, Kaprio J, Sarna S, Koskenvuo M. Relationship of leisure-time physical activity and mortality: the Finnish twin cohort. JAMA. 1998;279(6):440-4. Epub 1998/02/18. doi: 10.1001/jama.279.6.440. PubMed PMID: 9466636.
25. Leskinen T, Stenholm S, Heinonen OJ, Pulakka A, Aalto V, Kivimaki M, et al. Change in physical activity and accumulation of cardiometabolic risk factors. Prev Med. 2018;112:31-7. Epub 2018/04/02. doi: 10.1016/j.ypmed.2018.03.020. PubMed PMID: 29605421.
26. Tanasescu M, Leitzmann MF, Rimm EB, Willett WC, Stampfer MJ, Hu FB. Exercise type and intensity in relation to coronary heart disease in men. JAMA. 2002;288(16):1994-2000. Epub 2002/10/22. doi: 10.1001/jama.288.16.1994. PubMed PMID: 12387651.
27. Fogelholm M, Suni, J., Rinne, M., Oja, P., Vuori, I. Physical Activity Pie: A Graphical Presentation Integrating Recommendations for Fitness and Health. Journal of Physical Activity and Health. 2005;2:391-6.
28. Nagin DS, Odgers CL. Group-Based Trajectory Modeling (Nearly) Two Decades Later. J Quant Criminol. 2010;26(4):445-53. Epub 2010/12/07. doi: 10.1007/s10940-010-9113-7. PubMed PMID: 21132047; PubMed Central PMCID: PMCPMC2994902.
29. Jones BL ND. Traj - Group-based modeling of longitudinal data 1999 [updated 9/24/1999].
30. Park S, Lee JH. Associations of occupational and leisure-time physical activity with self-rated health in Korea. Prev Med. 2022;158:107022. Epub 20220317. doi: 10.1016/j.ypmed.2022.107022. PubMed PMID: 35307369.
31. Papp ME, Grahn-Kronhed AC, Rauch Lundin H, Salminen H. Changes in physical activity levels and relationship to balance performance, gait speed, and self-rated health in older Swedish women: a longitudinal study. Aging Clin Exp Res. 2022;34(4):775-83. Epub 20211116. doi: 10.1007/s40520-021-02016-5. PubMed PMID: 34784017; PubMed Central PMCID: PMCPMC9076716.
32. Hoertel N, Rico MS, Limosin F, Lemogne C, Alvarado JM, Goldberg M, et al. Retirement age does not modify the association of prior working conditions with self-rated health and mortality in retirees: results from a prospective study of retired French workers. Int Arch Occup Environ Health. 2022. Epub 20220610. doi: 10.1007/s00420-022-01886-0. PubMed PMID: 35687142.
33. Jylha M. What is self-rated health and why does it predict mortality? Towards a unified conceptual model. Soc Sci Med. 2009;69(3):307-16. Epub 20090610. doi: 10.1016/j.socscimed.2009.05.013. PubMed PMID: 19520474.
34. Bath PA. Self-rated health as a risk factor for prescribed drug use and future health and social service use in older people. J Gerontol A Biol Sci Med Sci. 1999;54(11):M565-70. doi: 10.1093/gerona/54.11.m565. PubMed PMID: 10619319.

For peer review only

Table 1. Pre-retirement characteristics of the study population (wave -1)

Variable	n	%
Gender		
Women	2,949	83
Men	601	17
Marital status		
Single	1,088	31
Co-habiting	2,462	69
ISCO class		
Professional	2,283	65
Manual or service worker	1,239	35
BMI		
BMI <30 kg/m^2	2,569	72
BMI >=30 kg/m^2	981	28
Smoking		
Not currently	3,192	91
Currently smoking	305	8.72
Alcohol consumption		
No risk-use	3,457	98
Risk-use	87	2
Suboptimal self-rated health		
No	2,690	76
Yes	853	24
Physical activity		
Low (<14 MET-h/week)	770	22
Moderate (14 to <30 MET-h/week)	638	18
High (≥30 MET-h/week)	2,142	60
	Mean	SD
Age	63.4	1.4

For peer review only

Table 2. Goodness of fit of group-based trajectory analysis models. The chosen model is shown in bold. Self-rated health treated as a binary variable.

Number of clusters	Shape of trajectory	Smallest group		BIC[1]	AIC[2]	Smallest APP[3]
		N	%			
1	Cubic	3,550	100	66,605	66,568	1.0
2	Cubic	861	24	64,626	64,552	0.94
**3**	**Cubic**	**308**	**9**	**62,956**	**62,845**	**0.93**
4	Cubic	64	2	62,434	62,286	0.89

[1] BIC = Bayesian Information Criterion, [2] AIC = Akaike information criterion, [3] APP = Smallest average posterior probability

For peer review only

Table 3. Risk ratios (RR) and their 95% confidence intervals (CI:s) of being classified to a certain trajectory group. RRs are adjusted for age and gender (only for age for gender RR). Statistically significant findings are shown in bold.

	Group #1			Group #2			Group #3		
**Variable/group**	**RR**	**95% CI**		**RR**	**95% CI**		**RR**	**95% CI**	
**Women vs. men**									
Group #1: Moderate physical activity and suboptimal self-rated health	1			0.82	0.66	1.01	1.29	0.94	1.78
Group #2: Moderate physical activity and good self-rated health	1.23	0.99	1.51	1			**1.59**	**1.19**	**2.11**
Group #3: increasing physical activity and improving self-rated health	0.77	0.56	1.07	**0.63**	**0.47**	**0.84**	1		
**Co-habiting vs. single**									
Group #1: Moderate physical activity and suboptimal self-rated health	1			**0.81**	**0.68**	**0.96**	**0.68**	**0.50**	**0.91**
Group #2: Moderate physical activity and good self-rated health	**1.23**	**1.04**	**1.47**	1			0.84	0.64	1.10
Group #3: increasing physical activity and improving self-rated health	**1.48**	**1.10**	**1.99**	1.20	0.91	1.57	1		
**Manual workers vs. professional workers**									
Group #1: Moderate physical activity and suboptimal self-rated health	1			**1.30**	**1.10**	**1.53**	**1.52**	**1.14**	**2.02**
Group #2: Moderate physical activity and good self-rated health	**0.77**	**0.65**	**0.91**	1			1.17	0.90	1.52
Group #3: increasing physical activity and improving self-rated health	**0.66**	**0.50**	**0.88**	0.86	0.66	1.11	1		
**BMI ≥30 vs. BMI <30**									
Group #1: Moderate physical activity and suboptimal self-rated health	1			**2.82**	**2.38**	**3.33**	**4.05**	**5.23**	**11.58**
Group #2: Moderate physical activity and good self-rated health	**0.35**	**0.30**	**0.42**	1			**2.76**	**1.88**	**4.05**
Group #3: increasing physical activity and improving self-rated health	**0.13**	**0.09**	**0.19**	**0.36**	**0.25**	**0.53**	1		
**Current smokers vs. former/never smokers**									
Group #1: Moderate physical activity and suboptimal self-rated health	1			**1.47**	**1.13**	**1.90**	**3.19**	**1.72**	**5.93**
Group #2: Moderate physical activity and good self-rated health	**0.68**	**0.52**	**0.88**	1			**2.18**	**1.20**	**3.95**
Group #3: increasing physical activity and improving self-rated health	**0.31**	**0.17**	**0.58**	**0.46**	**0.25**	**0.83**	1		
**Risk-users of alcohol vs. non-risk users**									
Group #1: Moderate physical activity and suboptimal self-rated health	1			**1.66**	**1.05**	**2.62**	**4.33**	**1.31**	**14.39**
Group #2: Moderate physical activity and good self-rated health	**0.60**	**0.38**	**0.96**	1			2.62	0.81	8.46
Group #3: increasing physical activity and improving self-rated health	**0.23**	**0.07**	**0.77**	0.38	0.12	1.24	1		

For peer review only

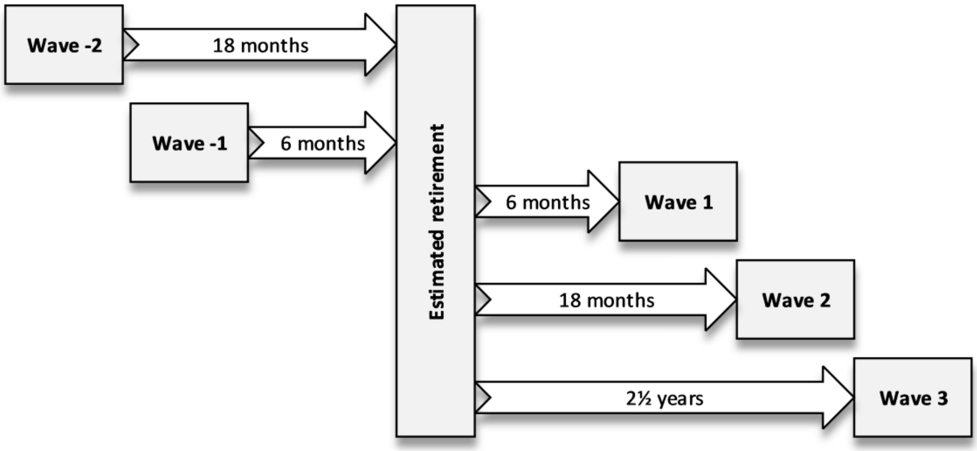

Figure 1. Study waves and time in relation to retirement

639x297mm (120 x 120 DPI)

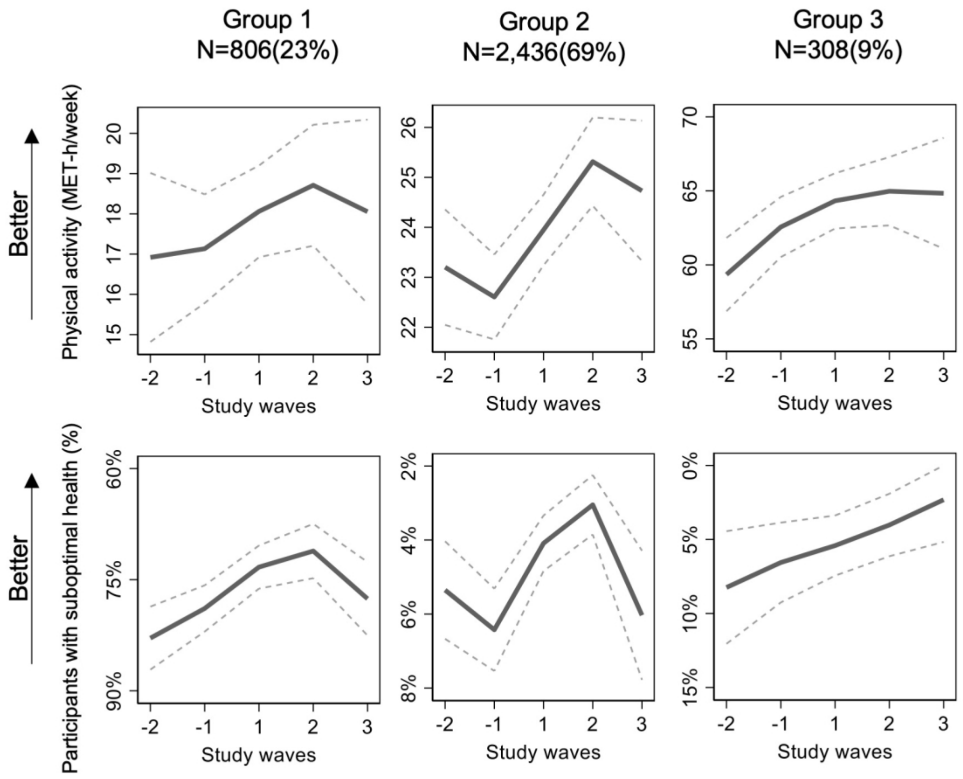

Figure 2. Trajectories of physical activity and self-rated health during retirement transition. The scale is different in each group.

139x117mm (330 x 330 DPI)

Supplement 1. Trajectories of physical activity and self-rated health. All the groups are presented within the same scale.

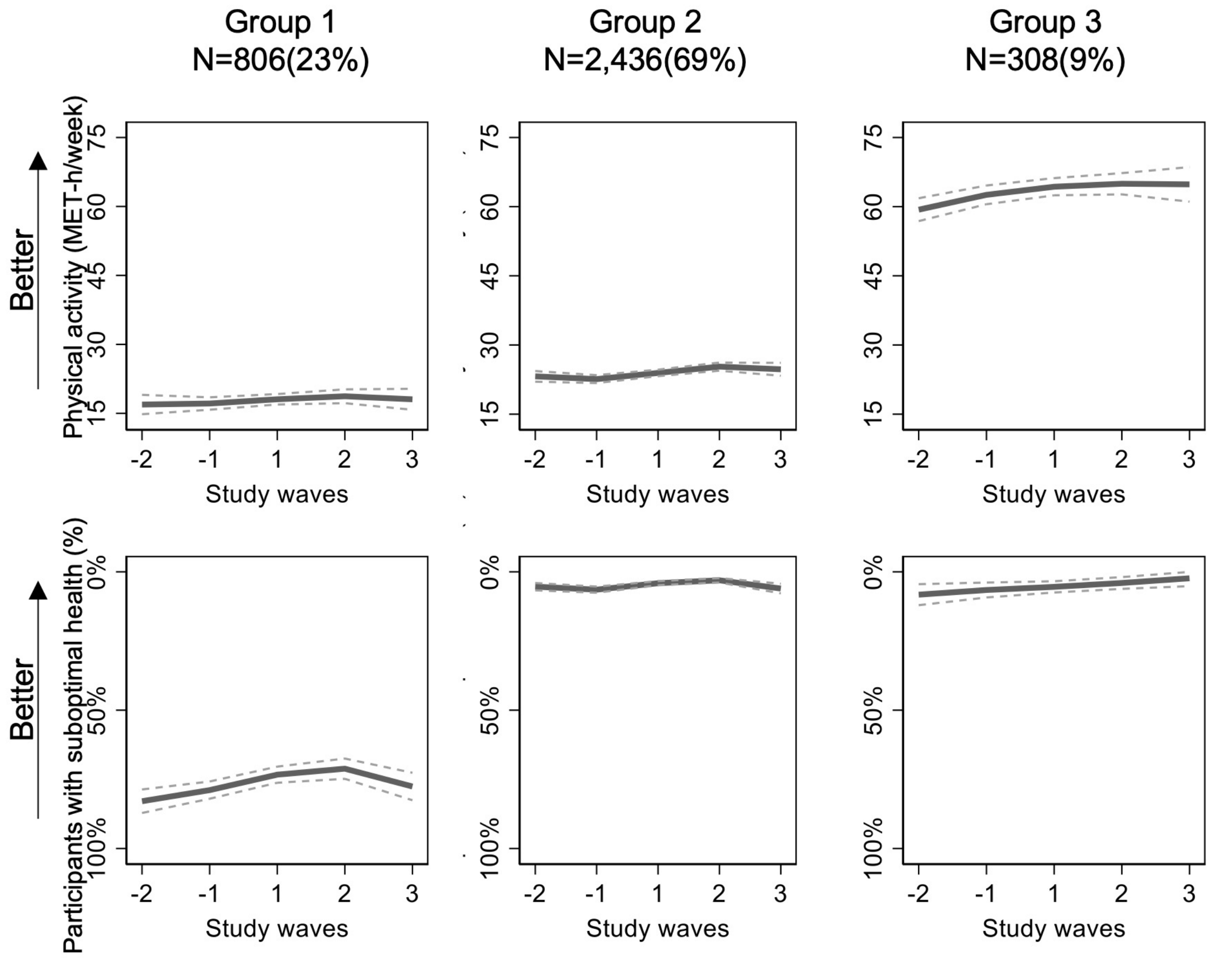

only

Supplement 2. The level of physical activity and physical functioning at different waves by trajectory groups

Study waves	Group #1:			Group #2:			Group #3:		
	Estimated mean	95% CI		Estimated mean	95% CI		Estimated mean	95% CI	
Physical activity, MET-h/week									
-2	16.9	14.8	19.0	23.2	22.1	24.4	59.4	56.9	61.8
-1	17.1	15.8	18.5	22.6	21.8	23.5	62.6	60.5	64.6
1	18.1	16.9	19.2	24.0	23.2	24.7	64.3	62.5	66.2
2	18.7	17.2	20.2	25.3	24.4	26.2	65.0	62.7	67.3
3	18.1	15.8	20.3	24.7	23.3	26.1	64.8	61.1	68.6
Percentage of participants with suboptimal self-rated health, %									
-2	83	79	87	5	4	7	8	4	12
-1	79	76	82	6	5	8	7	4	9
1	73	70	76	4	3	5	5	3	7
2	71	67	75	3	2	4	4	2	6
3	78	73	83	6	4	8	2	0	5

For peer review only

Supplement 3.

Trajectories of physical activity and self-rated health, self-rated health treated as a continuous variable. The 95% CI:s are presented as dash lines in the figure.

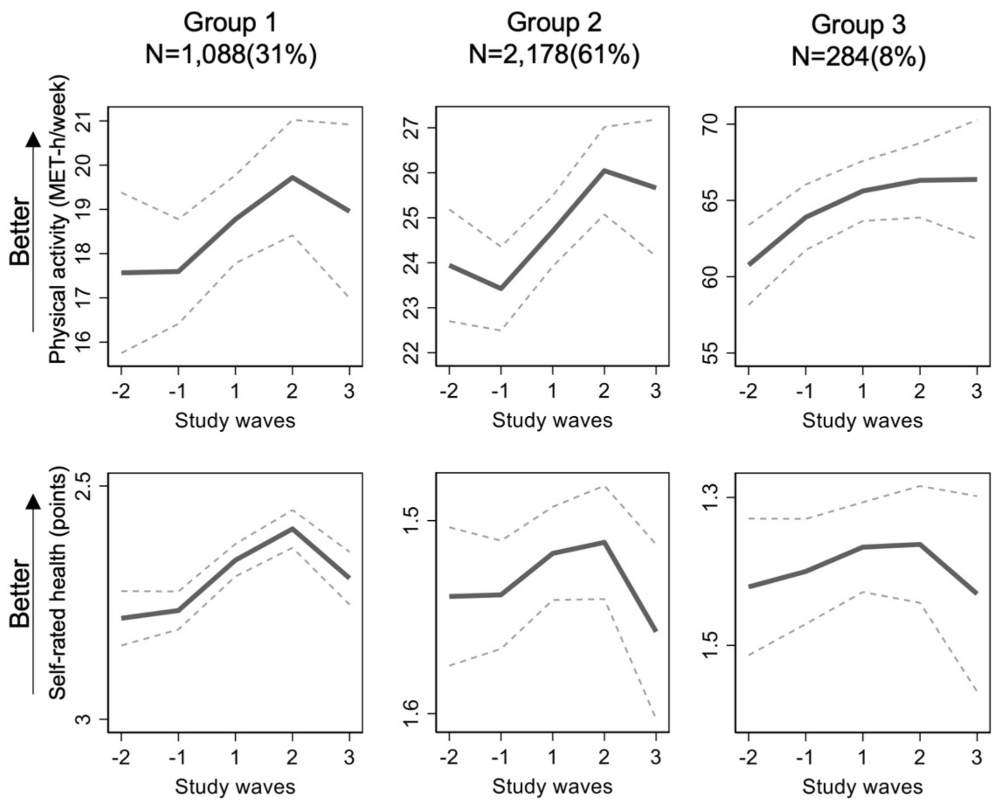

Goodness of fit of group-based trajectory analysis models, self-rated health treated as a continuous variable. The chosen model is shown in bold.

Number of clusters	Shape of trajectory	Smallest group		BIC[1]	AIC[2]	Smallest APP[3]
		N	%			
1	Cubic	3,550	100	75,810	76,769	1.0
2	Cubic	1,453	41	72,792	72,714	0.93
**3**	**Cubic**	**284**	**8**	**71,224**	**71,109**	**0.93**
4	Cubic	226	6	70,213	70,062	0.92

[1] BIC = Bayesian Information Criterion, [2] AIC = Akaike information criterion, [3] APP = Smallest average posterior probability

STROBE 2007 (v4) Statement—Checklist of items that should be included in reports of *cohort studies*

Section/Topic	Item #	Recommendation	Reported on page #
**Title and abstract**	1	(*a*) Indicate the study's design with a commonly used term in the title or the abstract	1
		(*b*) Provide in the abstract an informative and balanced summary of what was done and what was found	3
**Introduction**			
Background/rationale	2	Explain the scientific background and rationale for the investigation being reported	6,7
Objectives	3	State specific objectives, including any prespecified hypotheses	7
**Methods**			
Study design	4	Present key elements of study design early in the paper	8
Setting	5	Describe the setting, locations, and relevant dates, including periods of recruitment, exposure, follow-up, and data collection	
Participants	6	(*a*) Give the eligibility criteria, and the sources and methods of selection of participants. Describe methods of follow-up	8
		(*b*) For matched studies, give matching criteria and number of exposed and unexposed	
Variables	7	Clearly define all outcomes, exposures, predictors, potential confounders, and effect modifiers. Give diagnostic criteria, if applicable	8–10
Data sources/ measurement	8*	For each variable of interest, give sources of data and details of methods of assessment (measurement). Describe comparability of assessment methods if there is more than one group	8–10
Bias	9	Describe any efforts to address potential sources of bias	10,11, 17
Study size	10	Explain how the study size was arrived at	8
Quantitative variables	11	Explain how quantitative variables were handled in the analyses. If applicable, describe which groupings were chosen and why	8,9
Statistical methods	12	(*a*) Describe all statistical methods, including those used to control for confounding	10,11
		(*b*) Describe any methods used to examine subgroups and interactions	10,11
		(*c*) Explain how missing data were addressed	
		(*d*) If applicable, explain how loss to follow-up was addressed	
		(*e*) Describe any sensitivity analyses	11
**Results**			

For peer review only

Participants	13*	(a) Report numbers of individuals at each stage of study—eg numbers potentially eligible, examined for eligibility, confirmed eligible, included in the study, completing follow-up, and analysed	8
		(b) Give reasons for non-participation at each stage	
		(c) Consider use of a flow diagram	
Descriptive data	14*	(a) Give characteristics of study participants (eg demographic, clinical, social) and information on exposures and potential confounders	12, table 1
		(b) Indicate number of participants with missing data for each variable of interest	
		(c) Summarise follow-up time (eg, average and total amount)	
Outcome data	15*	Report numbers of outcome events or summary measures over time	12-14
Main results	16	(*a*) Give unadjusted estimates and, if applicable, confounder-adjusted estimates and their precision (eg, 95% confidence interval). Make clear which confounders were adjusted for and why they were included	12-14
		(*b*) Report category boundaries when continuous variables were categorized	8, 9
		(*c*) If relevant, consider translating estimates of relative risk into absolute risk for a meaningful time period	
Other analyses	17	Report other analyses done—eg analyses of subgroups and interactions, and sensitivity analyses	12-14
**Discussion**			
Key results	18	Summarise key results with reference to study objectives	15
**Limitations**			
Interpretation	20	Give a cautious overall interpretation of results considering objectives, limitations, multiplicity of analyses, results from similar studies, and other relevant evidence	15-17
Generalisability	21	Discuss the generalisability (external validity) of the study results	17
**Other information**			
Funding	22	Give the source of funding and the role of the funders for the present study and, if applicable, for the original study on which the present article is based	2

*Give information separately for cases and controls in case-control studies and, if applicable, for exposed and unexposed groups in cohort and cross-sectional studies.

Note: An Explanation and Elaboration article discusses each checklist item and gives methodological background and published examples of transparent reporting. The STROBE checklist is best used in conjunction with this article (freely available on the Web sites of PLoS Medicine at http://www.plosmedicine.org/, Annals of Internal Medicine at http://www.annals.org/, and Epidemiology at http://www.epidem.com/). Information on the STROBE Initiative is available at www.strobe-statement.org.

For peer review only
